# *Ent*–Clerodane Diterpenoid Inhibitors of Glucose-6-phosphatase from *Croton guatemalensis* Lotsy

**DOI:** 10.3390/plants15030442

**Published:** 2026-01-31

**Authors:** Sonia Marlen Escandón-Rivera, Adolfo Andrade-Cetto, Daniel Genaro Rosas-Ramírez, Gerardo Mata-Torres, Roberto Arreguín-Espinosa

**Affiliations:** 1 Departamento de Biología Celular, Facultad de Ciencias, Universidad Nacional Autónoma de México, Av. Universidad 3000, Circuito Exterior S/N, Coyoacán, Ciudad Universitaria, Mexico City 04510, Mexico; soniaer@ciencias.unam.mx (S.M.E.-R.); gerardom.torres@ciencias.unam.mx (G.M.-T.); 2 Departamento de Química de Biomacromoléculas, Instituto de Química, Universidad Nacional Autónoma de México, Av. Universidad 3000, Circuito Exterior S/N, Coyoacán, Ciudad Universitaria, Mexico City 04510, Mexico; dgrosas@unam.mx (D.G.R.-R.); arrespin@unam.mx (R.A.-E.)

**Keywords:** *Croton guatemalensis* Lotsy, Euphorbiaceae, *ent*–clerodane diterpenoids, G6Pase inhibitors, docking, HPLC-ESI-MS/MS profile

## Abstract

The Croton genus includes a diverse group of plants with remarkable potential in natural products research, particularly due to their bioactive compounds with hypoglycemic and phytochemical significance. This study examines *Croton guatemalensis* Lotsy, focusing on its chemical composition and its biological efficacy as a glucose-6-phosphatase inhibitor. Phytochemical analysis led to the isolation and structural elucidation of eleven compounds (**1**–**11**), including three new *ent*−clerodane diterpenes, designated crotoguatenoic acids C (**9**), D (**10**), and E (**11**). The absolute configurations of compounds **9**–**11** were determined by electronic circular dichroism (ECD) as (5*R,*8*R,*9*R,*10*S*)-configured *ent*–clerodanes. High-performance liquid chromatography–mass spectrometry (HPLC–MS/MS) revealed 25 peaks tentatively assigned to terpenoids, flavonoids, and alkaloids, highlighting the species’ chemical diversity. In vitro assays using ethanol–water extract (EWE) and isolated compounds with rat liver microsomes demonstrated inhibitory activity against glucose-6-phosphatase (G6Pase), particularly among *ent*–clerodane diterpenes (73–96%), with EWE and compounds **1**, **4**, and **11** showing the highest inhibition. Molecular docking analysis revealed strong interactions between these diterpenoids and the G6PC1 binding pocket, with binding energies comparable to chlorogenic acid (positive control). These findings position *C. guatemalensis* as a valuable source of bioactive diterpenoids and support the potential of *ent*-clerodane derivatives as natural G6Pase inhibitors for hyperglycemia management.

## 1. Introduction

The genus Croton (Euphorbiaceae), comprising over 1200 species distributed throughout tropical and subtropical regions, is well known for its diverse phytochemical profile. This includes diterpenoids, triterpenoids, alkaloids, flavonoids, and other phenolic compounds. Numerous pharmacological activities have been attributed to these metabolites, notably anti-inflammatory, antimicrobial, cytotoxic, and hypoglycemic effects [1,2]. Type 2 diabetes mellitus (T2D) remains a growing global health concern**.** Approximately 589 million adults (20–79 years) worldwide are estimated to live with diabetes, of which 43% (≈252 million) remain undiagnosed [3]. Projections estimate that the global number of diabetes cases will reach 853 million by 2050, with over 81% occurring in low- and middle-income countries. In 2024 alone, diabetes accounted for approximately 3.4 million deaths worldwide and imposed healthcare costs exceeding one trillion USD [3]. As the prevalence of type 2 diabetes continues to rise in regions where traditional medicine remains deeply rooted, the therapeutic use of medicinal plants has become an increasingly important component of diabetes management. These trends have spurred significant interest in plant-based therapeutics, especially those from traditional medicine, recognized as a rich source of structurally diverse and biologically active compounds [4].

*Croton guatemalensis* Lotsy, a small tree native to Central America and southern Mexico, is traditionally used to treat gastrointestinal disorders, skin infections, and diabetes [5,6]. Despite these ethnomedicinal uses, our group exclusively performed a comprehensive chemical analysis of the bark’s ethanol–water extract (EWE) [7], which led to the isolation of five *ent*–clerodane diterpenoids, including two novel structures (crotoguatenoic acids A and B) and three flavonoids (rutin, epicatechin, and quercetin). These compounds were fully characterized by NMR, ECD, and MS techniques, and affinity-directed fractionation revealed *ent*–clerodanes as *α*–glucosidase inhibitors. Given its traditional use as a hypoglycemic remedy by the Cakchiquel community in Guatemala and supported by prior in vivo studies showing its acute glucose-lowering effect [5], our research group performed mechanistic studies to elucidate the bioactivity of EWE and its primary constituent, junceic acid (**1**).

In a previous study, we explored possible mechanisms of action that could explain the hypoglycemic effect of *C. guatemalensis* extract and junceic acid. We demonstrated that, following administration of the extract and the compound in both fasting and postprandial states, insulin levels decreased in healthy and hyperglycemic rats despite reduced blood glucose concentrations in both metabolic states, suggesting a potential insulin-sensitizing effect. However, neither the compound nor the extract enhanced insulin action in insulin tolerance tests nor inhibited the activity of protein tyrosine phosphatase 1B, a negative regulator of the insulin signaling pathway. We also demonstrated that the extract and junceic acid inhibited the activity of two rate-limiting enzymes involved in hepatic glucose production. Compared with chlorogenic acid, junceic acid exerted a more potent inhibitory effect on glucose-6-phosphatase, which is commonly used as a positive control. We conclude that this species can modulate hyperglycemia primarily by reducing hepatic glucose production, specifically through inhibition of the rate-limiting enzyme G6Pase, while exerting a non-insulin-dependent sensitizing effect [8].

Building upon our previous findings, which identified junceic acid (**1**) as a novel clerodane–type diterpenoid with hypoglycemic activity via inhibition of G6Pase, we extended our investigation of the EWE from *C. guatemalensis* to isolate and characterize additional structurally related compounds that could also inhibit G6Pase. This targeted phytochemical approach led to the discovery and structural elucidation of three previously unreported *ent*–clerodane diterpenoids, providing an opportunity to evaluate their potential as G6Pase inhibitors. Concurrently, we conducted an untargeted HPLC–ESI–QTOF–MS/MS profiling of the extract, which revealed a diverse chemical composition including diterpenoids, alkaloids, and flavonoids. The isolated diterpenes were subsequently evaluated in vitro for their inhibitory activity against G6Pase, a key regulatory enzyme in gluconeogenesis and a validated therapeutic target for type 2 diabetes [9,10]. Remarkably, all three *ent*–clerodane diterpenoids exhibited inhibitory activity against G6Pase, underscoring the pharmacological relevance of this structural class within *C. guatemalensis*. To the best of our knowledge, this study represents the first to integrate comprehensive metabolomic profiling with G6Pase inhibitory evaluation in this species, thereby reinforcing its chemotaxonomic significance within the genus and highlighting its potential as a source of novel *ent*–clerodane-based scaffolds for hypoglycemic drug development.

## 2. Results and Discussion

**Figure 1:**
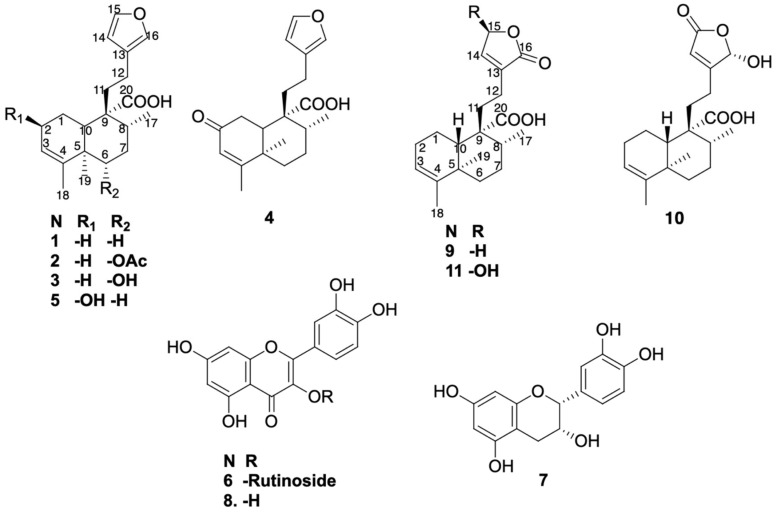
Isolated compounds from EWE of *C. guatemalensis*.

The isolated compounds (Figure 1) from the EWE of *C. guatemalensis*—eight *ent*–clerodane diterpenes (**1**–**5**, **9**–**11**) and three flavonoids (**6**–**8**)—were evaluated in vitro for their inhibitory activity against G6Pase, a key enzyme in hepatic glucose production. Junceic acid (**1**) had previously demonstrated in vivo hypoglycemic effects via this mechanism [8].

### 2.1. Structure Elucidation

A combination of ^1^H and ^13^C NMR spectral data (COSY, HSQC, HMBC, NOESY, TOCSY), high-resolution mass spectrometry (HRMS), and electronic circular dichroism experiments (ECDs) revealed the structures of three previously unreported compounds.

**Figure 2:**
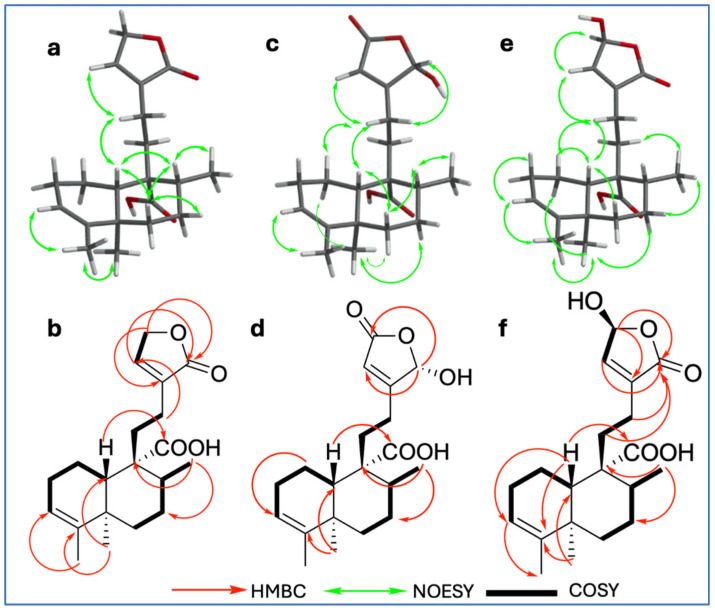
Key NOESY correlations of compounds **9** (**a**), **10** (**c**), and **11** (**e**); key HMBC (H—C) correlations of compounds **9** (**b**), **10** (**d**), and **11** (**f**); ^1^H-^1^H COSY correlations of compounds **9** (**b**), **10** (**d**), and **11** (**f**).

Compound **9** was isolated as a white powder with a melting point of 100–101 °C. Its molecular formula, C_20_H_28_O_4_, was established based on NMR spectroscopic data and confirmed by ESI-MS (HRESIMS) ion at *m*/*z* 331.19288 [M − H]^−^ (calcd. for C_20_H_27_O_4_, 331.19148), indicating seven degrees of unsaturation (Figures S1–S10). The IR spectrum (Figure S2) showed characteristic absorption bands for hydroxyl groups (broad band at 3387 cm^−1^), conjugated carbonyls (1745 cm^−1^ for a carboxylic acid and 1692 cm^−1^ for an ester), and olefinic functionalities (1448, 1406, 1375, and 1347 cm^−1^). The ^1^H, ^13^C, and DEPT NMR spectra (Table 1; Supplementary Data, Figures S3–S5) revealed a 20-carbon framework comprising three methyls, seven methylenes, two methines, two vinylic, and six quaternary carbons. The combined analysis of ^1^H and ^13^C data (Table 1, Figures S3 and S4), along with HSQC (Figure S6) and COSY (Figure S7) correlations, indicated the presence of a clerodane–type diterpenoid skeleton. The three methyl groups were identified as part of the core clerodane structure, with signals observed at *δ*_C/H_ 17.8/0.95 (s; C/H-19), 16.8/1.12 (d, *J* = 6.88 Hz; C/H-17), and 18.2/1.57 (q; *J =* 1.80 Hz C/H-18); COSY correlations between *δ*_H_ 4.78 (H-15) and 7.14 (H-14), along with HMBC correlations (Figure S8) from H-15 with *δ*_C_ 134.4 (C-13), 144.1 (C-14), and 174.0 (C-16), as well as from H-14 to *δ*_C_ 70.4 (C-15), 134.4 (C-13), and 174.3 (C-16), supported the presence of an *α*,*β*-unsaturated *γ*–lactone ring as a side chain moiety. A broad singlet at *δ*_H_ 5.23 (H-3) and its corresponding carbon signal at *δ*_C_ 121.3, along with *δ*_C_ 143.5 (C-4), indicated a trisubstituted double bond with no substitution at C-18. The carboxyl signal at *δ*_C_ 181.7 showed HMBC correlations with *δ*_H_ 1.61 (m, H-10) and *δ*_H_ 1.52 (m, H-8), confirming its location at C-20. The TOCSY (Figure S9) enables the unequivocal assignment of proton positions within the decalin system; thus, H-3 (*δ*_H_ 5.23) was found to be spin-coupled with *δ*_H_ 1.57 (H-18), 1.70 (m, H-1*α*), 1.90 (m, H-1*β*), and 2.00 (m, H-2*α* and H-2*β*), confirming its position within the A ring. Additionally, the proton at *δ*_H_ 1.52 (m, H-8) showed correlation with interaction with *δ*_H_ 1.19 (dd, H-6*α*), 1.78 (m, H-6*β*), 1.44 (dd, H-7*β*), 2.13 (dd, H-7*α*), and 1.61 (m, H-10), confirming its location within the B ring of the decalin system. The relative configuration of **9** was determined by NOESY spectroscopy (Figure S10). Key NOESY correlations (Figure 2a) were observed between H-6*β* (m, *δ*_H_ 1.78) and both H-10 (m, *δ*_H_ 1.61) and H-8 (m, *δ*_H_ 1.52), supporting their beta orientation; simultaneously, H-10 with H-12 (m, *δ*_H_ 2.23) and H-19 (s, *δ*_H_ 0.95) with H-7*α* (dd, *δ*_H_ 2.13), indicating a *trans*–*cis*–type configuration of the clerodane skeleton, which is characteristic of most of these diterpenes [11]. This configuration is consistent with the *ent*–clerodane structures previously reported by our group [7], although compound **9** shows distinct differences in the side chain.

**Table 1:** ^1^H and ^13^C NMR spectroscopy data of compounds **9**, **10**, and **11** (*δ* in ppm, *J* in Hz).

	9 ^a^		10 ^a^			11 ^a^
N	*δ*_H_	*δ*_C_	*δ*_H_	*δ*_C_	*δ*_H_	*δ*_C_
**1**	*α* 1.70, m *β* 1.90 ^b^, m	20.4	*α* 1.73, qd (12.21, 5.82) *β* 1.83 ^b^, m	20.4	*α* 1.70, m *β* 1.88 ^b^, m	20.4
**2**	*α* 2.00 ^b^, m *β* 2.00 ^b^, m	27.1	*α* 1.95 ^b^, m *β* 1.95 ^b^, m	27.4	*α* 2.03 ^b^, m *β* 2.03 ^b^, m	27.3
**3**	5.23, br s	121.3	5.25, br s	121.1	5.24, br s	121.2
**4**	-	143.5	-	143.5	-	143.5
**5**	-	38.9	-	38.9	-	38.9
**6**	*α* 1.19, dd (4.35, 13.35) *β* 1.78, m	37.5	*α* 1.20, dd (14.61, 5.16) *β* 1.82, m	37.4	*α* 1.21 ^b^, m *β* 1.79 ^b^, m	37.5
**7**	*α* 2.13, dd (13.01, 3.15) *β* 1.44, dd (13.75, 3.57)	27.5	*α* 2.11 ^b^, m *β* 1.45 ^b^, m	27.3	*α* 2.03 ^b^, m *β* 1.45 ^b^, m	27.4
**8**	1.52 ^b^, m	37.3	1.50, m	37.3	1.54 ^b^, m	37.2
**9**	-	49.9	-	49.8	-	49.9
**10**	1.61 ^b^, m	48.4	1.56 ^b^, m	48.5	1.59 ^b^, m	48.5
**11**	*α* 1.89 ^b^, m *β* 2.23 ^b^, m	31.5	*α* 1.91 ^b^, m *β* 2.27 ^b^, m	30.5	*α* 1.88 ^b^, m *β* 2.22 ^b^, m	31.2
**12**	2.23 ^b,c^, m	18.9	2.35 ^b,c^, m	21.1	2.03 ^b,c^, m	21.2
**13**	-	134.4	-	168.6	-	138.1
**14**	7.14, t (1.68)	144.1	5.90, br s	117.3	6.89, d (1.53)	143.4
**15**	4.78, d (1.89)	70.4	-	170.7	6.10, s	97.0
**16**	-	174.0	6.03, br s	98.7	-	171.7
**17**	1.12, d (6.88)	16.8	1.13, d (6.79)	16.7	1.12, d (6.85)	16.7
**18**	1.57, q (1.80)	18.2	1.59, br s	18.2	1.56, br s	18.2
**19**	0.95, s	17.8	0.96, s	17.7	0.94, s	17.7
**20**	-	181.7	-	180.5	-	181.2

^a^ Data recorded at 400 MHz (^1^H) and 150 MHz (^13^C) in CDCl_3_. ^b^ Overlapped signals. ^c^ Signals for two protons.

Compound **10** (C_20_H_28_O_5_) exhibited a close structural similarity to compound **9** (Table 1; Figures S11–S20), with the most notable difference being in the downfield shift observed in the chemical shifts corresponding to the side chain (C-13–C-16), which appeared downfield. Specifically, ^13^C and ^1^H NMR spectra showed a carbonyl at *δ*_C_ 170.7 (C-15) and a proton signal shifted downfield at *δ*_H_ 6.03 (br s, H-16) due to a hydroxyl at the position C-16, in contrast to the methine and carbonyl group arrangement found in compound **9**. The position of the carbonyl at C-15 and the hydroxyl group at C-16 was confirmed by the HMBC spectrum (Figure S18), which showed key correlations between H-14 (br s, *δ*_H_ 5.90) and C-13 (*δ*_C_ 168.6), C-15 (*δ*_C_ 170.7), and C-16 (*δ*_C_ 98.7). Additionally, H-16 (br s, *δ*_H_ 6.03) exhibited HMBC correlations with both C-13 and C-15, further supporting the proposed structural arrangement. The relative configuration at C-10 (*δ*_C_ 48.5) of compound **10** was determined by analysis of the NOESY spectrum (Figure S20); key NOESY correlations (Figure 2c) were observed between H-10 (m, *δ*_H_ 1.56) and both H-6*β* (m, *δ*_H_ 1.82) and H-12 (m, *δ*_H_ 2.35); simultaneously, correlations between H-19 (s, *δ*_H_ 0.98) with H-1*α* [*δ*_H_ 1.73, dd, *J =* qd (12.21, 5.82)] as well as H-7α (m, *δ*_H_ 2.11), supported a *trans*–*cis*–type clerodane skeleton configuration similar to compound **9.**

Compound **11** (C_20_H_28_O_5_) exhibited the same spectroscopic characteristics as **9** (Figures S21–S30). The only significant difference was observed in the downfield chemical shifts of C/H-15 (*δ*_C_ 97.0/*δ*_H_ 6.10, s), indicating the presence of a hydroxyl substituent at C-15. The relative configuration of **11** (Figure 2e) by NOESY NMR spectrum showed some key interactions between H-10 (m, *δ*_H_ 1.59) with H-6*β* (m, *δ*_H_ 1.79) and H-1*β* (m, *δ*_H_ 1.88); simultaneously, correlations between H-19 (s, *δ*_H_ 0.94) with H-1*α* (m, *δ*_H_ 1.70) as well as H-7*α* (m, *δ*_H_ 2.03) supported a *trans*–*cis*–type clerodane skeleton configuration similar to compounds **9** and **10**.

**Figure 3:**
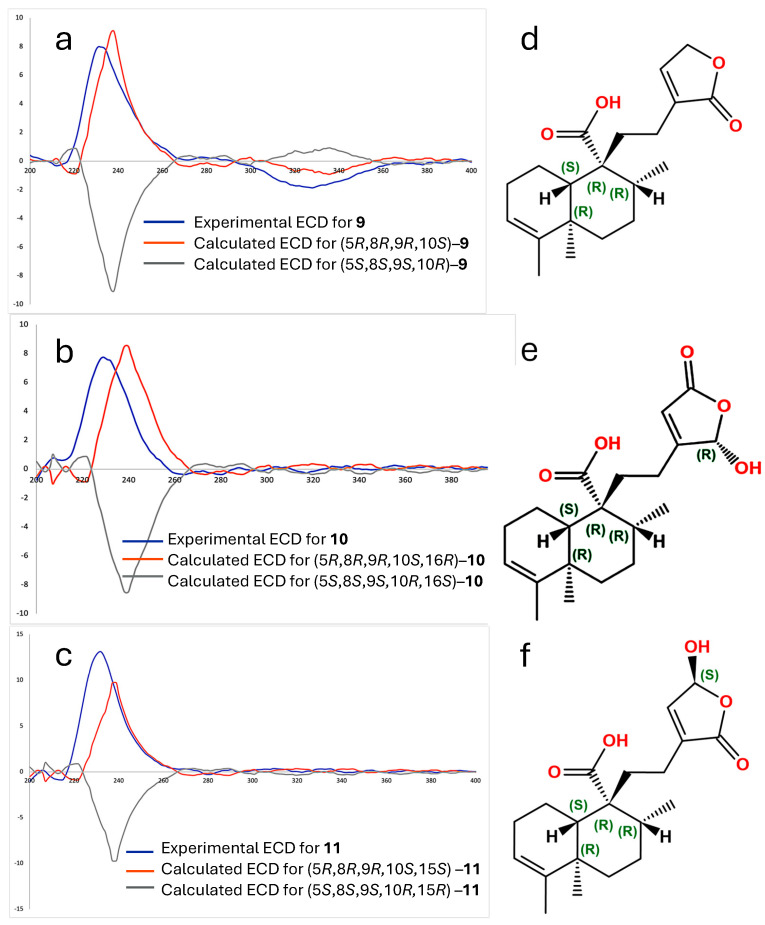
Experimental electronic circular dichroism (ECD) spectra for compounds **9** (**a**), **10** (**b**), and **11** (**c**), along with computed calculated CD spectra. Absolute configuration of the crotoguatenoic acids C (**d**), d (**f**) and g (**e**).

Specific rotation measurements and comparisons between experimental and calculated electronic circular dichroism (ECD) spectra for the *ent*–isomer were developed to establish the absolute configuration of compounds **9**, **10,** and **11** (Figure 3). The specific rotations were [α]^20^_D_ = −48, [α]^20^_D_ = −34, and [α]^20^_D_ = −36 for **9**, **10,** and **11**, respectively, showing all negative and of similar magnitude, suggesting a shared stereochemical framework with **9**. The experimental ECD spectra of compound **9** (blue line, Figure 3a), **10** (blue line, Figure 3b), and **11** (blue line, Figure 3c) exhibited Cotton effects that aligned well with those calculated for the *ent*–isomer (orange lines in Figure 2a and Figure 2c, respectively). Compounds **9**–**11** demonstrated positive Cotton Effects at 231 nm (Δε = +7.99), 232 nm (Δε = +13.10), and 229 nm (Δε = +7.75) and negative Cotton Effects at 328 nm (Δε = −1.87), 200 nm (Δε = −1.40), and 200 nm (Δε = −0.75), respectively. These spectral features closely matched the theoretical ECD profiles calculated for the (5*R*,8*R*,9*R*,10*S*) configuration. Therefore, the absolute configurations were confirmed as follows: (5*R*,*8R*,9*R*,10*S*)–*ent*–cleroda–3,13–dien–16,15–olide–20–oic acid (Figure 3d) for compound **9**; (5*R*,8*R*,9*R*,10*S*)–16–hydroxy–*ent*–cleroda–3,13-dien–15,16–olide–20–oic acid (Figure 3e) for compound **10;** and (5*R*,8*R*,9*R*,10*S*)–15–hydroxy–*ent*–cleroda–3,13–dien–16,15–olide–20–oic acid (Figure 3f) for compound **11**. Interestingly, the enantiomers of these structures were previously reported from the liverwort *Heteroscyphus coalitus* [12], where the isolated compounds showed positive optical rotation and mirror-image ECD spectra in comparison to compounds **9**–**11.** This confirms that the terpenoids isolated from *C. guatemalensis* are the opposite enantiomers of those previously described and further supports the *ent*–prefix assignment in their nomenclature. Following the naming convention of previously reported *ent*–clerodane diterpenes from *C. guatemalensis*, such as crotoguatenoic acids A (**2**) and B (**3**), these compounds have been designated as crotoguatenoic acids C, D, and E for compounds **9**, **10,** and **11**, respectively.

The clerodane diterpenes, identified in various plant species, are known to stereospecifically interact with biological targets, thereby influencing diverse biological activities. Most of them were evaluated as anti-cancer, anti-inflammatory, and antimicrobial [13]. The presence of opposite enantiomers in *C. guatemalensi* and *H. coalitus* suggests the potential for each to be exploited against different biological targets. While the *ent*–*neo*–clerodanes from *H. coalitus* have shown antifungal activity against *Candida albicans* [12], the *ent*–clerodanes isolated from *C. guatemalensis* may act through a distinct mechanism aligned with their traditional use. It would be valuable to evaluate both types of compounds in the same biological assays to expand our understanding and support the development of previously unreported phytopharmaceuticals. The difference in stereochemistry of these chemical profiles strongly suggests that these plants may have unique biological activities, emphasizing the importance of enantioselective isolation and characterization in natural product chemistry [14].

### 2.2. Qualitative Phytochemical Screening Profile of C. guatemalensis by HPLC–ESI–QTOF–MS/MS

The HPLC–ESI–QTOF–MS/MS profiles (Figure S34), utilizing the EWE in both positive and negative ionization modes (Table 2), were specifically developed to enhance the phytochemical characterization of *C. guatemalensis*. A total of 25 peaks were detected and tentatively identified as terpenes, flavonoids, alkaloids, and sucrose.

At the early retention times of the HPLC–ESI–QTOF–MS/MS profile (2–19 min), protonated molecules corresponding to typical flavonoid molecular formulas were observed, including derivatives of epigallocatechin, rutin, and isorhamnetin, often in glycosylated forms.

Flavanols are efficiently ionized in HPLC–ESI–QTOF–MS/MS and can be observed in both positive and negative ionization modes, depending on analytical objectives and the specific molecular structure. In positive ion mode, especially when using an acidic mobile phase, flavanols typically form protonated molecular ions [M + H]^+^ [18].

Several peaks observed at retention times of 2.72, 6.72, 6.95, and 9.40 min were tentatively assigned as flavan–3–ols. For example, the compound eluting at 6.72 min showed a deprotonated molecule at *m*/*z* 593.12832 [M − H]^−^ and a fragment ion at *m*/*z* 305.06658, consistent with the loss of a gallocatechin moiety (C_15_H_13_O_6_, 289.0712 Da). Similarly, the peaks at 2.72, 6.95, and 9.40 min showed the same fragmentation pattern, suggesting the similar functional groups [19]. These eluates exhibited typical procyanidin fragmentation patterns, including a characteristic fragment ion at *m*/*z* 139, which arises from C-ring cleavage through the 1,3-bond. This fragmentation behavior is consistent with the absence of substitution at the 3-position of the flavonoid skeleton and supports the presence of a flavanone [20]. In Table 2, the common fragmentation patterns of these flavanones can be observed.

Flavonols eluting at 12.3 and 18.7 min, with molecular formulas C_27_H_30_O_16_ and C_16_H_12_O_7_, showed protonated molecules at *m*/*z* 611.16333 [M + H]^+^ and 317.05280 [M + H]^+^, respectively. Positive ions at *m*/*z* 303.0362 and 303.0326 correspond to the loss of a rutinosyl moiety (C_12_H_20_O_9_) and a methyl group (CH_3_), respectively. These data are consistent with the presence of rutin (6) at 12.3 min and isorhamnetin at 18.7 min [18,21].

Isoquinoline alkaloids typically contain a basic nitrogen atom in their core structure, which readily undergoes protonation in solution and generates intense [M + H]^+^ signals [22]. Consequently, positive molecular ions of alkaloids were observed. Peaks at 5.35 and 5.90 min displayed protonated molecular ions at *m*/*z* 342.17248 [M + H]^+^ and 342.17277 [M + H]^+^, respectively, consistent with the molecular formula C_20_H_23_NO_4_. The MS/MS fragmentation patterns of both peaks matched those previously reported for isoquinoline alkaloids isolated from this genus [23]. The most informative product ions were [M + H − 17]^+^ or [M + H − 31]^+^, depending on whether the amino group is present as –NH_2_ or N-methylated (–N-CH_3_), reflecting neutral losses that are characteristic of aporphine-type alkaloids [24]. In this context, the peak at 5.35 min generated a fragment at *m*/*z* 293.10304 [M + H]^+^, indicative of a neutral loss of an N-methyl group, followed by loss of a hydroxyl substituent. Additionally, the overall fragmentation behavior closely resembled that of alkaloids such as isocorydine, previously identified in the genus Croton, suggesting that the alkaloid core skeleton is analogous to these known ones.

These isoquinoline alkaloids have been previously isolated from the ethanolic extract of *Croton linearis* [23] as well as from chloroform and dichloromethane extracts of *Croton hemiargyreus*, *C. echinocarpa*, *C. rivinifolius* [25], and *Croton lechleri* [26]. Corydine and some derivatives have been evaluated as G protein-biased full agonists of the opioid receptor (MOR), producing analgesic effects in a mouse model of visceral pain with minimal side effects. These properties make them very important in plants since they contribute to defense mechanisms and offer pharmacologically relevant scaffolds for drug development [27]. Nevertheless, corydine lacks in vivo or in vitro studies as hypoglycemic agents. However, isocorydine and some isoquinolin alkaloids were reported with hypoglycemic activity acting through inhibition of glucose uptake in intestinal membrane vesicles and reduced glucose absorption and blood glucose levels in vivo [28]. This information opens an interest in the isolation of these compounds from *C. guatemalensis* for their structural characterization and for their future studies as hypoglycemic agents.

The most prominent isolated constituents are terpenes bearing a clerodane-type skeleton. Based on this background, and considering that clerodane diterpenes are commonly found within the genus [29], we proposed molecular formulas for the remaining unidentified peaks. Fifteen peaks were classified as diterpenes. Compounds of this nature began eluting at 19.4 min and continued up to 33.4 min. Some of them (at 21.4, 25.5, 25.9, 27.1, 27.9, 28.3, 29.6, and 33.4 min) were identified by comparing their retention times and mass spectra with those of previously isolated compounds (**1**–**5**, **9**, **10**, and **11**). Other peaks presented similar molecular formulas to those of isolated compounds. For example, the peak at 17.7 min, with negative ion at *m*/*z* 347.18694 [M − H]^−^, exhibited the same molecular formula as crotoguatenoic acids D and E (C_20_H_28_O_5_). On the other hand, peaks at 18.5, 19.4, 21.0, 24.6, and 30.0 min, detected as positive or negative ions at 379.17493 [M − H]^−^, 365.19766 [M + H]^+^, 347.18789 [M + H]^+^, 389.19891 [M + H]^+^, and 393.22698 [M + H]^+^, respectively, exhibited fragmentation patterns similar to those of known clerodane diterpenes. In particular, the peak at 30.0 min showed fragment ions at *m*/*z* 315, 145, 133, 131, 119, and 117, closely matching the fragmentation behavior reported for crotoguatenoic acid B, which supports a similar structural nature. Taken together, these data and the corresponding molecular formulas are consistent with a clerodane diterpene skeleton bearing different substituents, such as methyl or hydroxyl groups. Based on these observations, we propose that these peaks also correspond to clerodane diterpenes, similar to those previously isolated. In this context, *C. guatemalensis* suggests a complex chemical profile consisting of diterpenes and flavonoids. The presence of diterpenes and flavonoids draws a parallel with the phytochemical profile reported for other species in the genus, such as *C. lechleri* and *C. heliotropiifolius* [2]. Reinforcing the chemotaxonomic significance of these metabolite classes in Croton.

### 2.3. Evaluation of Inhibition of Glucose-6-phosphatase (G6pase)

EWE and all the isolated compounds were tested for G6Pase inhibition, using CA as a positive control. Table 3 presents the inhibition percentages in descending order, along with their respective IC_50_ (µg/mL). All the *ent*–clerodane diterpenes and EWE inhibited the enzyme (73–96%). Crotoguatenoic acid E (**11**) exhibited the highest activity, achieving 96% inhibition, closely approaching the 99% inhibition of the positive control, CA. Junceic acid (**1**) and crotoguatenoic acids C (**9**) and D (**10**) were the other compounds that demonstrated at least 80% inhibition of G6Pase enzymatic activity. In contrast, rutin (**6**) showed weak inhibition and reduced enzymatic activity by only 37% (Table 3). Therefore, the three isolated flavonoids are unlikely to be responsible for the EWE activity. The clerodanes displayed similar inhibition (Figure S36). Based on their IC_50_ values (Table 3), the clerodanes’ activity decreased in the following order: formosin F (**4**), junceic acid (**1**), crotoguatenoic acid B (**3**), E (**11**), A (**2**), D (**10**), C (**9**), and bartsiifolic acid (**5**), with corresponding IC_50_ values of 484.3 ± 97.1, 579 ± 91.9, 655.3 ± 53.2, 772.3 ± 80.3, 828.5 ± 16.5, 943.3 ± 161.9, 1081.3 ± 202.9, and 1275 ± 257.3 µg/mL, respectively. This increase or decrease demonstrates the importance of a functional group present or absent in the structure; for example, compound **4** displayed a similar IC_50_ to CA, even better than **1**, and these only structurally differ by a carbonyl in the C-2 position. Conversely, compound **5**, which has a hydroxyl at the C-2 position instead of a carbonyl, exhibited the lowest activity among all tested clerodane diterpenes. This suggests that the carbonyl group, particularly its *α*,*β*-unsaturation, is crucial for enhancing enzyme affinity, possibly by acting as a Michael acceptor and complexing with nucleophilic protein residues at either the catalytic site or allosterically. These are biologically active molecules with low levels of toxicity [30,31]. Like compound **5**, compound **3** also undergoes hydroxylation at the C-6 position; however, unlike compound **5,** it demonstrates greater activity, comparable to that of junceic acid (**1**). There is much evidence of increased inhibitory activity of clerodane diterpenes in different targets related to the presence of a hydroxyl at the C-6 position, but this activity is diminished when this hydroxyl is replaced by an acetyl or methoxyl [11,13,32], which coincides for this assay in the case of compound **2**, which exhibits lower activity, possibly due to some steric hindrance. The furan ring represents another significant functional group in these structures, appearing to be more influential than the presence of a *γ*-lactone, as observed in compounds **9**, **10**, and **11**. The orientation of the furan ring influences activity; specifically, compound **11**, with the *γ*-lactone carbonyl at C-16, shows greater activity compared to compound **10**, which has the carbonyl at C-15. Ultimately, the EWE exhibited the best IC_50_ value, surpassing even that of CA. Given the inhibitory activity demonstrated by all *ent*–clerodanes, they likely complement one another, which could result in a synergistic effect [33]. These findings are in agreement with prior in vivo studies demonstrating that both EWE and junceic acid (**1**) exert hypoglycemic effects by suppressing hepatic glucose output, specifically via inhibition of the rate-limiting enzyme G6Pase [8]. Collectively, these data reinforce the pharmacological rationale for the traditional use of *C. guatemalensis* in diabetes management.

G6Pase is a key enzyme in the final step of both gluconeogenesis and glycogenolysis. It catalyzes the conversion of glucose–6–phosphate to free glucose and is an essential process for maintaining blood glucose levels, especially during fasting [10]. Since G6Pase enzymatic activity is elevated in patients with T2D compared to healthy individuals [34], inhibiting or reducing this activity can help manage hyperglycemia associated with the disease. Crotoguatenoic acids (A to E), formosin F (**4**), and bartsiifolic acid (**5**) have shown promise in inhibiting G6Pase. Therefore, these structures serve as promising models for treating T2D by reducing hepatic glucose production.

### 2.4. Molecular Docking

**Figure 4:**
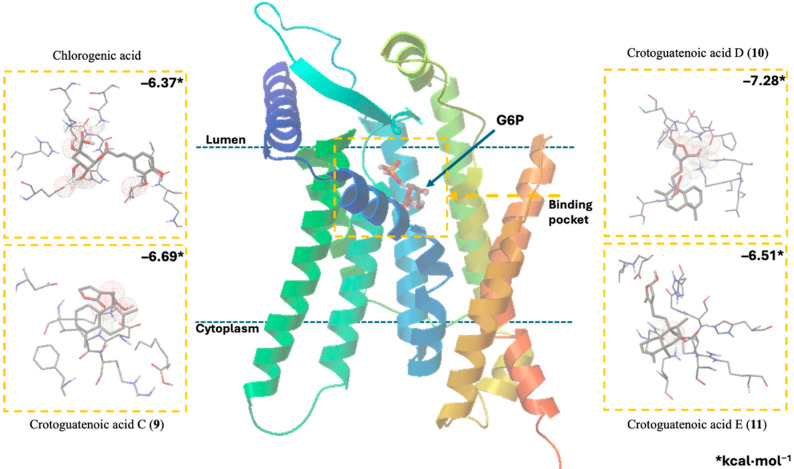
Docking results using the structural model of the ligand–receptor molecular model for human G6PC1 (9J7V.PDB) with G6P, CA, and compounds **9** to **11**.

Docking studies were conducted to elucidate the interaction of the compounds within the pocket of the human G6PC1 crystal structure (9J7V.PDB), which served as the molecular modeling template [35]. To test the adapted protocol with G6PC1 templates [35,36], ligand–receptor molecular models were also calculated for CA (positive control) and glucose-6-phosphate (G6P). G6P interacts with the amino acid His176, which is the most important residue in the catalytic pocket of the analyzed enzyme (Figure 4, Table 4) [35]. It forms hydrogen bonds between the amino acid residues Lys76 (1.714 and 1.771 Å), Thr111 (1.663 and 1.881 Å), Pro116 (2.07 Å), Gly118 (2.196 Å), and His176 (1.905 Å). CA fits better than G6P (Figure 4, Table 4), forming hydrogen bond interactions between the amino acid residues Asp38 (1.73 Å), Asn72 (2.133 Å), Lys76 (1.849 Å), Glu110 (2.16 and 1.913 Å), Gly118 (2.156 Å), and His176 (2.044 Å).

The ligand–receptor calculations for the *ent*–clerodane diterpenoids **1**–**5** and **9**–**11** were well-fitted in the catalytic pocket of the analyzed enzyme (Figure 4, Table 4). Compound **1** showed hydrogen bonding interactions between the amino acid residues Leu39 (2.174 Å), Asn72 (1.945 Å), Lys76 (1.839 Å), and Gly118 (2.207 Å). Compound **2** showed hydrogen bonding interactions between the amino acid residues Lys76 (2.176 Å), Thr111 (2.043 Å), and Ser260 (1.902 Å). Compound **3** showed hydrogen bonding interactions between the amino acid residues Lys76 (1.868 Å) and His176 (2.016 Å). Compound **4** showed hydrogen bonding interactions between the amino acid residues Lys76 (1.933 Å), Thr111 (1.774 Å), Gly118 (2.057), and His176 (2.158 Å). Compound **5** showed hydrogen bonding interactions between the amino acid residues Val107 (2.075 Å), Arg170 (1.957 Å), and His252 (1.951 Å). Compound **9** showed hydrogen bonding interactions between the amino acid residues Leu39 (2.108 Å) and Lys263 (2.121 Å). Compound **10** showed hydrogen bonding interactions with the amino acid residues Asn72 (2.219 Å), Lys76 (1.85 and 1.893 Å), Pro116 (2.15 Å), Gly118 (2.075 Å), and His176 (1.881 Å). Similarly, compound **11** showed hydrogen bonding interactions with the amino acid residues Asn72 (2.181 Å) and Lys76 (1.839 Å).

Recent studies that provided information on the structure, substrate recognition, and catalytic mechanism of G6Pase [35] enable molecular recognition studies to be conducted with this enzyme complex [36]. These studies help suggest the possible mechanism of action of these hypoglycemic agents as G6Pase inhibitors, as well as the structural requirements for the biological activity of these bioactive metabolites. G6Pase comprises nine transmembrane helices and possesses a large catalytic pocket facing the lumen [35]. Docking studies allowed us to observe how diterpenes cause steric hindrance by interacting with the amino acid residue His176, which is a crucial part of G6P recognition [35].

**Figure 5:**
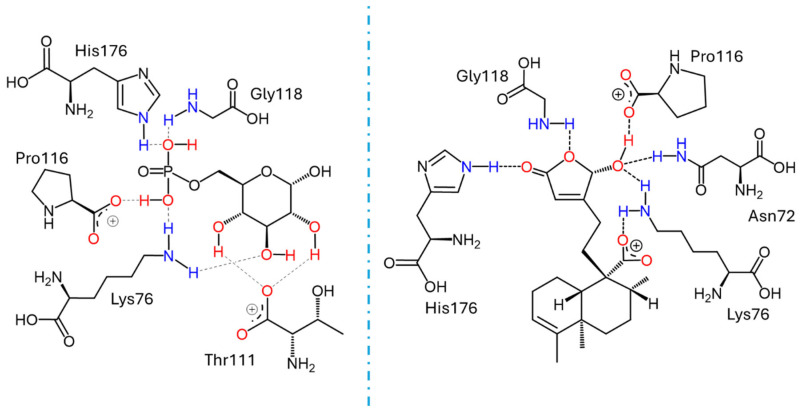
Docking results for the human G6PC1 (9J7V.PDB) structural model, encompassing the catalytic site and the binding interactions for G6P with the residues Lys76, Thr111, Pro116, Gly118, and His176 (**left**); and for crotoguatenoic acid D (**10**) with the residues Asn72, Lys76, Pro116, Gly118, and His176 (**right**).

**Figure 6:**
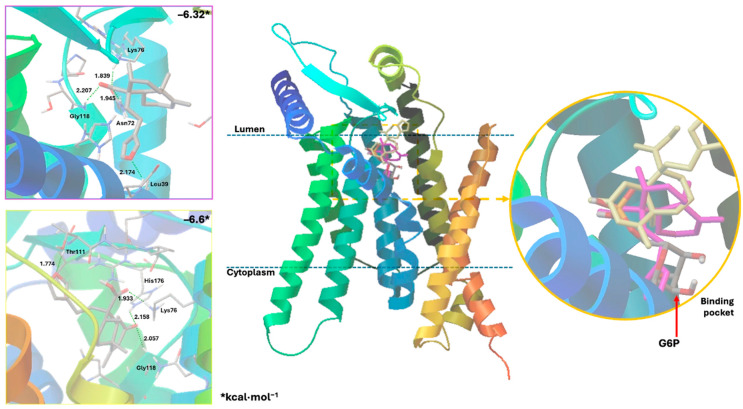
Docking results using the structural model of the ligand–receptor molecular model for human G6PC1 (9J7V.PDB) with **1** (pink) and **4** (yellow).

These results suggest that crotoguatenoic acid D (**10**) represents the optimal conformation among the *ent*–clerodane diterpenoids for inhibiting the studied enzyme (Table 4), as evidenced by a lower dissociation constant *K*_i_ for G6PC1 (4.64 μM) and a lower binding energy (ΔG_binding_ = −7.28 kcal/mol). Compound **10** binds to the enzyme’s catalytic site in a specific conformation and orientation that creates a significant steric block at the receptor surface. This steric hindrance effectively prevents access to the catalytic pocket (Figure 5). Interestingly, junceic acid (**1**) and formosin F (**4**) exhibited binding energies comparable to CA (Figure 5, Table 4). Junceic acid (**1**) showed four hydrogen bonds with the residues Leu39 (2.174 Å), Asn72 (1.945 Å), Lys76 (1.839 Å), and Gly118 (2.207 Å), displaying a binding energy (ΔG_binding_ = −6.32 kcal/mol) and a dissociation constant (*K*_i_ = 23.46 mM). In the case of formosin F (**4**), similar to compound **1**, it formed four hydrogen bonds with the residues Lys76 (1.933 Å), Thr111 (1.774 Å), Gly118 (2.057 Å), and His176 (2.158 Å), but with slightly better binding energy (ΔG_binding_ = −6.6 kcal/mol) and dissociation constant (*K*_i_ = 14.42 μM) than the values obtained for **1**, CA, and G6P (Figure 6, Table 4) and interacting with the catalytic amino acid His176 [35] with a hydrogen bond. These results were observed both experimentally in the in vitro assay and theoretically in the docking assay (Table 3 and Table 4, Figure 6). Figure 6 shows that, in addition to the furan ring and carboxylic acid functional groups, the *α*,*β*-unsaturated carbonyl of **4** could interact with the most important residue, His176. Since **1** does not exhibit this interaction, the earlier hypothesis in Section 2.3 is supported.

With docking analysis, four very important functional groups were observed in the structures: the carboxylic acid, the furan ring, the *α*,*β*-unsaturated carbonyl, and the *α*,*β*-unsaturated gamma–lactone. These interact with protein residues through different bond formations between the functional group and the protein. *α*,*β*-unsaturated carbonyls are Michael acceptors that can generate covalent bonds with nucleophilic protein residues such as cysteine (Cys), histidine (His), and lysine (Lys) [31]; gamma–lactones form non-covalent bonds such as hydrophobic forces and hydrogen bonds with hydrophobic residues (such as leucine (Leu), isoleucine (Ile), valine (Val), phenylalanine (Phe)) and polar residues (e.g., serine, threonine, tyrosine), in addition to possible interactions with polar side groups or the peptide backbone [37]. Furans can form covalent adducts mainly with lysine (Lys), but also, under certain conditions, with cysteine (Cys) and tyrosine (Tyr) [38]; finally, carboxylic acids interact through hydrogen and salt bonds with basic residues (Lys, Arg) and rarely covalently [39]. These functionalities collectively position *ent*–clerodanes as promising G6Pase inhibitors.

Driven by the recent interest in clerodanes isolated from Croton species for T2D treatment, there has been an increased evaluation of these structures against different biological targets. For example, fifteen compounds isolated from *Croton yunnanensis* were tested for their ability to promote glucose uptake in insulin-resistant 3T3-L1 adipocytes, thus acting as insulin sensitizers [40]. In the same way, eighteen clerodanes from *Croton mangelong* were tested for hypoglycemic activity in insulin-resistant 3T3-L1 adipocytes, and ten of them demonstrated activity. Moreover, mangelonine D demonstrated a significant hypoglycemic effect (30 mg/kg) in a streptozotocin-induced hyperglycemic Sprague–Dawley rat model [41]. Conversely, junceic acid (**1**) from *C. guatemalensis* demonstrated a hypoglycemic effect, reducing postprandial insulin levels in healthy and hyperglycemic rats, suggesting an insulin-sensitizing effect [8]. Collectively, these investigations underscore the potential of clerodane diterpenes as promising candidates for diabetes treatment.

## 3. Conclusions

The phytochemical investigation of *C. guatemalensis* led to the isolation of several clerodane-type diterpenes, including the novel crotoguatenoic acids C–E (compounds **9**–**11**), whose absolute configurations were confirmed by ECD.

The HPLC–ESI–MS/MS analysis proposed the presence of diterpenes, flavonoids, and alkaloids in *C. guatemalensis*. Notably, the alkaloids in this species are identified for the first time in this species, highlighting a novel phytochemical component. This finding underscores the need for targeted isolation and characterization of these alkaloids to evaluate their potential as hypoglycemic agents.

In patients with type 2 diabetes, hepatic glucose production is overactive, driven by both gluconeogenesis and glycogenolysis. Targeting glucose-6-phosphatase (G6Pase) allows simultaneous modulation of both pathways, thereby reducing hepatic glucose output and improving fasting hyperglycemia.

In vitro biological evaluation of the isolated compounds from *C. guatemalensis* revealed that the *ent*–clerodane diterpenes inhibit G6Pase, with inhibition levels reaching up to 96%. Comparative analysis suggests a structure–activity relationship influenced by functional group substitutions at C-15 and C-16 of the clerodane core, as well as variations at C-2 and C-6. Specifically, the presence of an *α*,*β*-unsaturated carbonyl group at the C-2 position of the decalin system enhances inhibitory activity, whereas substitution with a hydroxyl group diminishes it. Docking analyses further indicate that a *γ*–lactone moiety at C-15 and C-16 may confer greater activity than a furan ring at the same positions.

These findings establish *C. guatemalensis* as a promising natural source of bioactive *ent*–clerodane diterpenes with potential applications in managing hyperglycemia. Further evaluations and mechanistic studies are essential to validate their therapeutic potential as G6Pase inhibitors.

## 4. Materials and Methods

### 4.1. General

Analytical and semi-preparative high-performance liquid chromatography (HPLC) experiments were conducted using an Agilent 1260 Infinity system (Agilent Technologies, Santa Clara, CA, USA). This system includes a G1311B quaternary pump, a G1367E autosampler, and a G1315C diode-array detector (DAD VL+), with data acquisition and processing via ChemStation software (Agilent Technologies, Santa Clara, CA, USA). Analytical separations were achieved using a Luna Omega Polar C18 column (50 × 2.1 mm, 1.6 μm particle size; Phenomenex, Torrance, CA, USA). For semi-preparative purposes, a Nucleosil C18 column (Macherey-Nagel; 250 × 10 mm, 5 μm) was also employed. Rutin, epicatechin, and quercetin reference compounds (purity ≥ 94% by HPLC) were procured from Sigma-Aldrich (St. Louis, MO, USA). Column chromatography was performed using Sephadex LH-20 (Sigma-Aldrich) or silica gel 70–230 mesh (Merck Mexico). Thin-layer chromatography (TLC) was performed on silica gel 60 F254 plates (Macherey-Nagel, Düren, Germany), and spots were visualized using a 10% ceric sulfate solution in sulfuric acid.

A Varian Inova 400 MHz spectrometer (Varian Inova, Palo Alto, CA, USA), operating at 400 MHz for ^1^H and 150 MHz for ^13^C nuclei, acquired nuclear magnetic resonance (NMR) spectra, including one-dimensional (^1^H, ^13^C, DEPT) and two-dimensional (HSQC, HMBC, COSY, NOESY, TOCSY) experiments. Chemical shift values are expressed in parts per million (*δ*, ppm). High-resolution electrospray ionization mass spectrometry was performed using an Agilent HPLC-ESI-QTOF system (Model G6530BA; Agilent Technologies system, Santa Clara, CA, USA). Additional high-resolution mass spectrometry (HR-MS) data were recorded with a JEOL AccuTOF JMS-T100LC instrument (HR-DART-MS; JEOL USA, Peabody, MA, USA). Electronic circular dichroism (ECD) and Fourier-transform infrared (FT-IR) spectra were measured using a J-1500 spectropolarimeter (JASCO, Oklahoma City, OK, USA) and a Tensor 27 instrument (Bruker Optics), respectively.

### 4.2. Plant Material and Extracts

*Croton guatemalensis* was collected in 2019 from the Department of Chimaltenango, Guatemala, by Dr. Carola Cruz, based on prior ethnobotanical research [5]. A specimen was deposited at the Deshidrafarmy-Farmaya Herbarium with the voucher CFEH 1259.

EWE was prepared by extracting 20 g of dried *C. guatemalensis* material twice using 500 mL of a 1:1 ethanol–water mixture for 4 h each time, resulting in a total extract volume of 1 L. The combined extracts were filtered and concentrated at 40 °C with a Büchi Labortechnik AG rotary vacuum evaporator (Flawil, Switzerland). The aqueous residue was subsequently lyophilized, yielding 4.864 g of dry extract, which was stored at 4 °C for further studies. For the phytochemical study, the same methodology previously reported by our group [7] was applied, using a larger amount of plant material. Dried and ground *C. guatemalensis* (120.7 g) was extracted with a 1:1 ethanol–water mixture (6 L) over 4 h. The extract was then filtered and sequentially partitioned with dichloromethane (CH_2_Cl_2_; 3 × 6 L), followed by ethyl acetate (EtOAc; 3 × 6 L). This process yielded 11.4871 g of CH_2_Cl_2_-soluble fraction (DSF), 2.6885 g of EtOAc-soluble fraction (ESF), and 11.5303 g of water-soluble fraction (WSF).

### 4.3. Compounds Isolation

This study aimed to determine whether structurally related diterpenoids and co-occurring flavonoids contribute to the overall activity of the extract. To obtain additional quantities of the previously isolated compounds from the EWE of *C. guatemalensis*, the extract was subjected to repeated silica gel column chromatography, following previously reported methodology [7]. As a result, compounds **1**–**8** were isolated as well as three previously undescribed *ent*–clerodane diterpenes (**9**–**11**) (Figure 1).

DSF (10.6 g) was partitioned onto 378 g of silica gel (70–230 mesh, Merck Mexico) using column chromatography. An eluent system of n–hexane, ethyl acetate (EtOAc), and methanol (MeOH) was used, starting with 100% n–hexane and gradually increasing the polarity with EtOAc and then MeOH. Following TLC analysis of their chromatographic profiles, 133 collections were obtained (each 100 mL). This process led to 37 primary fractions (DSF1-DSF37), respectively. Fractions DSF1 (1.364 g) and DSF4 were obtained as the pure compounds **1** (junceic acid) and **2** (crotoguatenoic acid A), respectively. Preparative TLC of fractions DSF8 (79.0 mg; CH_2_Cl_2_:MeOH, 96:4; 1.0 mm), DSF9 (116.4 mg; CH_2_Cl_2_:MeOH, 96:4; 1.0 mm), and DSF11 (95.0 mg CHCl_3_:MeOH, 95:5; 1.0 mm) yielded 15.2 mg of **3** (crotoguatenoic acid B), 25.2 mg of **5** (bartsiifolic acid), 33.5 mg of **4** (formosin F), 36.6 mg of **9**, and 22.8 mg of **11**. The semi-preparative HPLC separation of 50 mg of DSF10 was conducted using a mixture of 15:85 MeCN:H_2_O as the mobile phase for 15 min (2.0 mL/min; 240 nm UV-detection) to obtain 15.0 mg of **10** (Rt = 5.5 min) and 16.1 mg of **4** (Rt = 6.4 min).

Using MeOH as the eluent, ESF (2.37 g) was subjected to 100 g of Sephadex LH-20, resulting in 89 subfractions (ESF1–ESF89). They were injected into the HPLC-DAD to obtain the fractions for rutin (**6**) and epicatechin (**7**). Conditions of the HPLC profile were performed following the methodology previously developed by our group [7]. Elution was carried out at a flow rate of 0.35 mL/min using solvent A (water with 0.1% formic acid) and solvent B (acetonitrile, MeCN) under the following gradient program: 99:1 (A:B) at 0 min, 80:20 at 14 min, 50:50 from 14 to 26 min, 70:30 from 26 to 34 min, 20:80 from 34 to 35 min, and returning to 99:1 from 35 to 38 min. The column temperature was 35 °C. The UV detection was monitored at 254 and 365 nm. Compound **6** (rutin) was obtained as a pure isolate (49 mg) from fraction ESF25. Fraction ESF14–ESF17 (87 mg) was further purified by semi-preparative HPLC using a Nucleosil C18 column (250 × 10 mm internal diameter, 5 μm; Macherey-Nagel). Elution was performed with a 10:90 mixture of MeCN:H_2_O (*v*/*v*) at a flow rate of 2.0 mL/min over 15 min, with UV detection at 280 nm, affording compound **7** (epicatechin) as a pure compound (25 mg, Rt = 9.0 min).

#### 4.3.1. (5R,8R,9R,10S)-Ent-cleroda-3,13-dien-16,15-olide-20-oic Acid (Crotoguatenoic Acid C; **9**)

White powder; [α]^20^_D_ = −48 (c 0.001 MeOH); UV (MeOH) λ_max_ (log ε) 214 (1.937) nm; ECD (MeOH) λ_max_ (Δε) 231 (+7.99), 328 (−1.87); IR *ν*_max_ 3387 (OH), 1745 (COOH), 1692 (C=O), 1448 (C=C), 1375 (C=C), 1347 (C=C) cm^−1^; ^1^H NMR (CDCl_3_, 400 MHz), and ^13^C NMR (CDCl_3_, 150 MHz), see Table 1; HRESIMS 331.19288 [M − H]^−^ (calcd. for C_20_H_27_O_4_, 331.19148).

#### 4.3.2. (5R,8R,9R,10S)-16-Hydroxy-ent-cleroda-3,13-dien-15,16-olide-20-oic Acid (Crotoguatenoic Acid D; **10**)

White powder; [α]^20^_D_ = −34 (c 0.001 MeOH); ECD (MeOH) (Δε) 232 (+13.10), 200 (−1.40); IR νmax 3380 (OH), 1697 (C=O), 1561 (C=C), 1382 (C=C) cm^−1^; ^1^H NMR (CDCl_3_, 400 MHz) and ^13^C NMR (CDCl_3_, 150 MHz), see Table 1; ESI–MS *m*/*z* 695.6 [2M − H]^−^, HRESIMS ion at *m*/*z* 349.20157 [M + H]^+^ (calcd. for C_20_H_29_O_5_, 349.20204).

#### 4.3.3. (5R,8R,9R,10S)-15-Hydroxy-ent-cleroda-3,13-dien-16,15-olide-20-oic Acid (Crotoguatenoic Acid E; **11**)

White powder; [α]^20^_D_ = −36 (c 0.001 MeOH); ECD (MeOH) (Δε) 229 (+7.75), 200 (−0.75); IR νmax 3424 (OH), 1695 (C=O), 1765 (C=O),1453 (C=C), 1381 (C=C) cm^−1^; ^1^H NMR (CDCl_3_, 400 MHz) and ^13^C NMR (CDCl_3_, 150 MHz), see Table 1; HRESIMS *m*/*z* 347.15660 [M − H]^−^ (calcd. for C_20_H_27_O_5_, 347.18639).

### 4.4. Instrumental and Chromatographic Conditions for HPLC–QTOF–ESI–MS/MS Analysis

High-resolution mass spectra were acquired using high-performance liquid chromatography coupled with quadrupole time-of-flight mass spectrometry (HPLC–QTOF–ESI–MS/MS) on an Agilent 1260 Infinity LC system equipped with a G6530BA QTOF mass spectrometer (Agilent Technologies, USA). To identify the bioactive constituents present in EWE of *C. guatemalensis*, the extract and compounds were dissolved in deionized water and methanol, respectively, and 3 μL aliquots were injected into a Poroshell C18 column (5 × 3.0 mm i.d., 2.6 μm particle size) maintained at 35 °C for analysis. Chromatographic separation was performed using a binary gradient elution system with solvent A (acetonitrile) and solvent B (deionized water) at a flow rate of 0.5 mL/min. The gradient program was as follows: 99:1 (A:B) at 0 min, 80:20 at 14 min, 50:50 from 14 to 26 min, 70:30 from 26 to 34 min, and 20:80 from 34 to 35 min. Mass spectrometric conditions included the following optimized parameters: electrospray ionization (ESI) in positive and negative mode, ion spray voltage at 3.5 kV, fragmentor voltage at 80 V, and both drying and sheath gas temperatures at 350 °C. The sheath gas flow rate was set at 10 L/min. Accurate mass measurements were obtained for all protonated molecules.

All detected ions were recorded in high-resolution mode, and molecular formulas were assigned based on the comparison between experimental and calculated exact masses. Retention times observed *m*/*z* values, and reference ions were used to annotate the compounds. A comparative analysis of the fragmentation patterns of each peak was carried out against public spectral libraries. Specifically, the observed analyte ions were searched in MassBank (MassBank of mass spectral data, MassBank Consortium, available at https://massbank.eu, accessed on 2 January 2025) and GNPS in order to tentatively annotate the detected features. Previously reported phytochemical profiles of the species and published HPLC-DAD chromatographic data for this plant additionally guided the proposed molecular structures, providing a consistent framework for assigning likely metabolite classes. The identification process was further supported by literature data on previously reported metabolites in related Croton species.

### 4.5. Computational Details

This process was carried out exactly as previously described by our group [7]. Spartan’14 software generated energy-minimized structures for all ligands via geometry optimization using the semiempirical PM3 method. Resulting conformers were filtered for redundancy and subsequently optimized using B3LYP/DGDZVP density functional theory (DFT) implementation in Gaussian 09 software. At this level, thermochemical parameters, infrared (IR) spectra, and vibrational frequency analyses were also computed. For the major conformers in methanol, TD-SCF calculations were performed using the same functional and basis set along with the default solvent model to obtain theoretical circular dichroism (TCD) spectra. The calculated excitation energies (in nm) and corresponding rotatory strengths (R) in dipole velocity form (R_vel_) were used to simulate TCD spectra via the Harada–Nakanishi equation, as implemented in SpecDis version 1.71.

### 4.6. Evaluation of Inhibition of Glucose-6-phosphatase (G6Pase)

To prepare the G6Pase enzyme, subcellular fractions were isolated from the livers of Wistar rats. Two Wistar rats were fasted for 18 h prior to the procedure. The animals were then anesthetized with pentobarbital (6 mg/100 g body weight, administered intraperitoneally). After anesthesia, the livers were dissected and homogenized in buffer containing 250 mM sucrose, 1 mM EDTA, and 5 mM HEPES (pH 7.4). The homogenate was subjected to differential centrifugation to obtain the desired fractions. The resulting pellets were stored at −40 °C until further use [42]. After resuspending the obtained microsomal fraction in buffer (250 mM sucrose, 40 mM imidazole, pH 7), different concentrations (2 µg/mL to 5.0 mg/mL) of inhibitor samples (CA or compounds) were added to the reaction mixture. The enzymatic reaction was started by adding 80 mM G6Pase and incubating at 20 °C for 20 min. The reaction was stopped by adding a “stop solution”, which consists of 0.42% ammonium molybdate in 1 N H_2_SO_4_, 10% SDS, and 10% ascorbic acid. After that, it was incubated at 45 °C for 20 min. Absorbance was measured at 830 nm [43].

### 4.7. Docking Calculations

Docking was performed using Auto Dock 4.2 software (The Scripps Research Institute, La Jolla, CA, USA) with default parameters as previously described [44,45]. Crystal structure resolution of the human wild-type (WT) G6PC1 apo form (9J7V.PDB) was used for docking calculations. The calculations for the energy-minimized form with geometric optimization for all ligands were developed with HyperChem 8. Protein and ligand protonation states were assigned according to AutoDockTools 4.2 defaults at physiological pH. Polar hydrogens were added, non-polar hydrogens were merged into the enzyme structures, and Gasteiger charges were calculated for the molecular models of the analyzed compounds, following the procedure previously described for G6PC1 [35,36]. All file preparations were performed using AutoDockTools 4.2. Surface scanning and refined docking were performed to analyze the binding modes accurately. To refine these results, the best conformations observed for the ligand–receptor molecular models in the preliminary analysis were docked into a smaller area to analyze the binding modes to G6PC1. In the refined docking, the grid map was a 40 × 40 × 40 grid point. The Lamarckian genetic algorithm was applied. During the docking experiment, 100 independent runs per ligand were carried out. Auto Dock 4.2 software was used for obtaining the theoretical inhibition constant (*K*_i_) by the binding energy (∆G) obtained using the formula *K*_i_ = exp(∆G/RT), where T is the temperature (298.15 °K) and R is the universal gas constant (1.985 × 10^−3^ kcal mol^−1^ K^−1^).

## Figures and Tables

**Table 2:** Compounds tentatively identified from EWE by HPLC-ESI-QTOF-MS/MS.

N	t_R_ (min)	Ion Mode (Negative)	Ion Mode (Positive)	Molecular Formula	Exact Mass (Calcd Error, ppm)	Fragmentation Pattern	Plausible Identification
1	0.53	341.11190 [M − H]^−^	381.08060 [M + K]^+^	C_12_H_22_O_11_	381.07991 (1.7)	101.0745, 113.0269	Sucrose
2	2.72	-	443.09488 [M + H]^+^	C_22_H_18_O_10_	443.09837 (−7.8)	159.0609, 131.0615, 117.0523, 115.0495, 105.06628, 103.0496.	Epicatechin gallate (ECG) [15]
3	4.78	-	386.20267 [M + H]^+^	C_22_H_27_NO_5_	386.19729 (13)	346.16045, 327.19338, 325.18961, 279.14635, 223.1079, 179.0913, 178.0893, 150.0961, 130.0426, 129.059, 122.065, 113.0832, 112.082.	(1,2,9,10-Tetramethoxy-6-methyl-5,6,6a,7-tetrahydro-4H-dibenzo[de,g]quinolin-3-yl)methanol
4	5.35	-	342.17248 [M + H]^+^	C_20_H_23_NO_4_	342.16998 (7.2)	293.10304, 271.11984, 205.0999, 146.0725, 142.0585.	Isocorydin [16]
5	5.90	-	342.17277 [M + H]^+^	C_20_H_23_NO_4_	342.16998 (8.2)	300.121, 282.119, 224.1116, 205.09, 178.0895, 167.0735, 157.0698, 122.0655.	Isocorydin isomer [16]
6	6.72	593.12832 [M − H]^−^	-	C_30_H_26_O_13_	593.13006 (−2.9)	426.7677, 305.06658, 234.8924, 136.8934.	Gallocatechin-(4α → 8)-catechin [15]
7	6.95	-	307.08456 [M + H]^+^	C_15_H_14_O_7_	307.08232 (7.2)	177.0458, 163.0319, 159.0368, 149.0527, 145.0215, 139.0328, 135.0374, 117.0282.	Epigallocatechin [15]
8	9.40	289.07256 [M − H]^−^	291.07320 [M + H]^+^	C_15_H_14_O_6_	291.08686 (2.0)	265.0734, 237.08, 219.07, 191.0756, 147.0373, 139.0325, 123.0384.	Epicatechin (**7**) [7]
9	11.3	-	223.06300 [M + H]^+^	C_11_H_10_O_5_	223.06010 (12)	207.0186, 190.0266, 179.0256, 163.0279, 135.03363, 134.0301, 107.0444, 106.0366, 105.0288.	Isofraxidin
10	12.3	609.14627 [M − H]^−^	611.16333 [M + H]^+^	C_27_H_30_O_16_	609.146657 (1.6)	303.0362, 129.0482, 127.0322	Rutin (**6**) [7]
11	17.7	347.18694 [M − H]^−^	-	C_20_H_28_O_5_	347.18639 (1.5)	318.8871, 275.466, 275.3352, 275.1882, 275.1258, 274.9379.	Unknown clerodane diterpene *
12	18.5	379.17493 [M − H]^−^	-	C_20_H_28_O_7_	379.17622 (−3.0)	299.742, 288.9046, 271.7683, 254.9546, 190.9616.	Unknown clerodane diterpene *
13	18.7	315.05070 [M − H]^−^	317.05280 [M + H]^+^	C_16_H_12_O_7_	315.051575 (4.1)	303.0326, 301.0209, 274.0356, 178.1147, 166.1151, 136.0773, 135.0746, 119.0805, 110.0533.	Isorhamnetin [17]
14	19.4	-	365.19766 [M + H]^+^	C_20_H_28_O_6_	365.29696 (1.9)	253.1465, 239.1317, 211.1279, 197.088, 175.1038, 173.0909, 169.0932, 135.0743, 119.0811, 105.0651.	Unknown clerodane diterpene *
15	21.0	-	347.18789 [M + H]^+^	C_20_H_26_O_5_	347.18639 (4.3)	301.1671, 243.1265, 145.0946, 136.0779, 135.0746, 133.0951, 119.08, 111.0417.	Unknown clerodane diterpene *
16	21.4	347.18737 [M − H]^−^	-	C_22_H_28_O_5_	347.18639 (2.8)	301.9047, 274.9253, 272.9253, 257.9246, 236.9225, 220.9447, 204.9431, 175.9601.	Crotoguatenoic acid D
17	24.6	-	389.19891 [M + H]^+^	C_22_H_28_O_6_	389.19696 (5.0)	318.2874, 275.661, 257.2564, 230.2383, 107.0844.	Unknown clerodane diterpene *
18	25.5	329.18937 [M − H]^−^	331.19365 [M + H]^+^	C_20_H_26_O_4_	331.19148 (6.0)	203.1344, 183.1082, 159.1091, 145.0942, 135.074, 133.1091, 121.0957, 119.0803, 107.081, 105.0655.	Formosin F (**4**) [7]
19	25.9	331.18785 [M − H]^−^	-	C_20_H_28_O_4_	331.19148 (10)	329.8909, 285.9441, 283.9294, 257.9723, 255.9552, 221.0402, 107.9436.	Bartsiifolic acid (**5**) [7]
20	27.1	331.19288 [M − H]^−^	-	C_20_H_28_O_4_	331.19148 (4.2)	330.87, 302.908.	Crotoguatenoic acid C (**9**)
21	27.9	347.18705 [M − H]^−^	-	C_20_H_28_O_5_	347.18639 (1.5)	331.19303, 311.8724, 296.8681, 220.9505, 183.8435.	Crotoguatenoic acid E (**11**) [7]
22	28.3	-	333.20824 [M + H]^+^	C_20_H_28_O_4_	333.20171 (3.3)	315.19778, 287.20276, 145,0942, 135.1099, 133.0387, 131.0792, 121.0952, 119.0798, 117.0807, 105.065.	Crotoguatenoic acid B [7]
23	29.6	373.20347 [M − H]^−^	397.1999 [M + H]^+^	C_22_H_30_O_5_	373.20204 (3.4)	315.1817, 311.8724, 296.9263, 269.1783, 286.9263, 258.9589, 202.9505, 183.8435.	Crotoguatenoic acid A [7]
24	30.0	-	393.22698 [M + H]^+^	C_22_H_32_O_6_	393.22826 (−3.4)	315.1809, 279.2191, 205.1854, 184.0654, 147.1097, 145.0944, 133.0944, 131.0795, 119.0802, 117.0642, 107.0802, 105.0653.	Unknown clerodane diterpene *
25	33.4	315.19777 [M − H]^−^	317.21272 [M + H]^+^	C_20_H_28_O_3_	315.19656 (3.8)	314.8966, 310.873, 271.9509, 183.845, 148.9563.	Junceic acid [7]

* unknown: compound that has no prior record in the literature.

**Table 3:** Inhibition of G6Pase by EWE and isolated compounds.

	% of Inhibition	IC_50_ (µg/mL) ±SD
Chlorogenic acid (control)	99	406.7 ± 2.3
Crotoguatenoic acid E (**11**)	96	772.3 ± 80.3
EWE	90	301 ± 80
Junceic acid (**1**)	84	579 ± 91.9
Crotoguatenoic acid C (**9**)	83	1081.3 ± 202.9
Crotoguatenoic acid D (**10**)	80	943.3 ± 161.9
Formosin F (**4**)	79	484.3 ± 97.1
Crotoguatenoic acid B (**3**)	78	655.3 ± 53.2
Crotoguatenoic acid A (**2**)	73	828.5 ± 16.5
Bartsiifolic acid (**5**)	73	1275 ± 257.3
Epicatechin (**7**)	56	3899 ± 156.5
Rutin (**6**)	37	NA

**Table 4:** G6PC1 theoretical inhibition of diterpenoid selected for docking study.

	Binding Energy	Inhib Constant	Hydrogen Bond
G6P	−4.23	792.59 mM	Thr111, 1.663 Gly118, 2.196 Thr111, 1.881 His176, 1.905 Lys76, 1.714 Pro116, 2.07 Lys76 1.771
CA	−6.37	21.29 mM	Asp38, 1.73 Glu110, 2.16 His119, 2.044 Lys76, 1.849 Asn72, 2.133 Glu110, 1.913 Gly118, 2.156
**1**	−6.32	23.46 mM	Asn72 1.945, Gly118, 2.207 Lys76, 1.839 Leu39, 2.174
**2**	−6.68	12.59 mM	Lys76, 2.176 Ser260, 1.902 Thr111, 2.043
**3**	−6.12	32.88 mM	Lys76, 1.868 His176, 2.016
**4**	−6.6	14.42 mM	Thr111, 1.774 Gly118, 2.057 Lys76, 1.933 His176, 2.158
**5**	−5.7	66.6 mM	Val107, 2.075 His252, 1.951 Arg170, 1.957
**9**	−6.69	12.43 mM	Leu39, 2.108 Lys263, 2.121
**10**	−7.28	4.64 mM	Lys76, 1.893 Gly118, 2.075 Lys76, 1.85 His176, 1.881 Pro116, 2.15 Asn72, 2.219
**11**	−6.51	16.8 mM	Asn72, 2.181 Lys76, 1.839

## Data Availability

The original contributions presented in this study are included in the article/Supplementary Material. Further inquiries can be directed to the corresponding author.

## Supplementary Materials

The following supporting information can be downloaded at: https://www.mdpi.com/article/10.3390/plants15030442/s1, Figure S1: HRESI-MS spectrum of compound **9**; Figure S2: IR spectrum of compound **9**; Figure S3: ^1^H-NMR spectrum of compound **9** (400 MHz, CDCl_3_); Figure S4: ^13^C-NMR spectrum of compound **9** (125 MHz, CDCl_3_); Figure S5: DEPT spectrum of compound **9** (135 MHz, CDCl_3_); Figure S6: HSQC spectrum of compound **9** (400 MHz, CDCl_3_); Figure S7: COSY spectrum of compound **9** (400 MHz, CDCl_3_); Figure S8: HMBC spectrum of compound **9** (400 MHz, CDCl_3_); Figure S9: TOCSY spectrum of compound **9** (400 MHz, CDCl_3_); Figure S10: NOESY spectrum of compound **9** (400 MHz, CDCl_3_); Figure S11. HRESI-MS spectrum of compound **10**; Figure S12: IR spectrum of compound **10**; Figure S13: ^1^H-NMR spectrum of compound **10** (400 MHz, CDCl_3_); Figure S14: ^13^C-NMR spectrum of compound **10** (125 MHz, CDCl_3_); Figure S15: DEPT spectrum of compound **10** (135 MHz, CDCl_3_); Figure S16: HSQC spectrum of compound **10** (400 MHz, CDCl_3_); Figure S17: COSY spectrum of compound **10** (400 MHz, CDCl_3_); Figure S18: HMBC spectrum of compound **10** (400 MHz, CDCl_3_); Figure S19: TOCSY spectrum of compound **10** (400 MHz, CDCl_3_); Figure S20: NOESY spectrum of compound **10** (400 MHz, CDCl_3_); Figure S21: HRESI-MS spectrum of compound **11**; Figure S22: IR spectrum of compound **11**; Figure S23: ^1^H-NMR spectrum of compound **11** (400 MHz, CDCl_3_); Figure S24: ^13^C-NMR spectrum of compound **11** (125 MHz, CDCl_3_); Figure S25: DEPT spectrum of compound **11** (135 MHz, CDCl_3_); Figure S26: HSQC spectrum of compound **11** (400 MHz, CDCl_3_); Figure S27: COSY spectrum of compound **11** (400 MHz, CDCl_3_); Figure S28: HMBC spectrum of compound **11** (400 MHz, CDCl_3_); Figure S29: TOCSY spectrum of compound **11** (400 MHz, CDCl_3_); Figure S30: NOESY spectrum of compound **11** (400 MHz, CDCl_3_); Figure S31: Experimental circular dichroism spectrum of **9**; Figure S32: Experimental circular dichroism spectrum of **10**; Figure S33: Experimental circular dichroism spectrum of **11**; Figure S34: Phytochemical screening profile of *C. guatemalensis* by HPLC–ESI–Q–TOF–MS/MS in positive and negative modes; Figure S35. MS/MS spectra of the HPLC-MS/MS profile of the ethanol:water extract of *C. guatemalensis*; Figure S36: Concentration–response inhibition curves of G6Pase.

## Abbreviations

The following abbreviations are used in this manuscript:

ECD	Electronic circular dichroism
HPLC–MS	High-performance liquid chromatography–mass spectrometry
CA	Chlorogenic acid
G6Pase	glucose-6-phosphatase
T2D	Type 2 diabetes
HRMS	high-resolution mass spectrometry
