# Supplementary material for: Ent–Clerodane Diterpenoid Inhibitors of Glucose-6-phosphatase from Croton guatemalensis Lotsy
Source: Plants (Basel). 2026 Jan 31;15(3):442. doi: 10.3390/plants15030442 (PMC12899306; doi:10.3390/plants15030442)
Supplement: Supplementary file 1 [file plants-15-00442-s001.zip › plants-4008900-supplementary.pdf]

Ent–clerodane diterpenoid inhibitors of glucose-6-phosphatase from *Croton guatemalensis* Lotsy.

Sonia Marlen Escandón-Rivera[1], Adolfo Andrade-Cetto[1*], Daniel Genaro Rosas-Ramírez[2], Gerardo Mata-Torres[1] and Roberto Arreguín-Espinosa[2]

1 Departamento de Biología Celular, Facultad de Ciencias, Universidad Nacional Autónoma de México, Av. Universidad 3000, Circuito Exterior S/N, Coyoacán, Ciudad Universitaria, Mexico City, 04510; soniaer@ciencias.unam.mx, gerardom.torres@ciencias.unam.mx, aac@ciencias.unam.mx

2 Departamento de Biomacromoléculas, Instituto de Química, Universidad Nacional Autónoma de México, Av. Universidad 3000, Circuito Exterior S/N, Coyoacán, Ciudad Universitaria, Mexico City, 04510; dgrosas@unam.mx, arrespin@unam.mx

* Correspondence: aac@ciencias.unam.mx; Tel.: +525556225437

Figure S1. HRESI-MS spectrum of compound **9.**

Figure S2. IR spectrum of compound **9.**

Figure S3. ^{1}H-NMR spectrum of compound **9** (400 MHz, $CDCl_3$).

Figure S4. ^{13}C-NMR spectrum of compound **9** (125 MHz, $CDCl_3$).

Figure S5. DEPT spectrum of compound **9** (135 MHz, $CDCl_3$).

Figure S6. HSQC spectrum of compound **9** (400 MHz, $CDCl_3$).

Figure S7. COSY spectrum of compound **9** (400 MHz, $CDCl_3$).

Figure S8. HMBC spectrum of compound **9** (400 MHz, $CDCl_3$).

Figure S9. TOCSY spectrum of compound **9** (400 MHz, $CDCl_3$).

Figure S10. NOESY spectrum of compound **9** (400 MHz, $CDCl_3$).

Figure S11. HRESI-MS spectrum of compound **10.**

Figure S12. IR spectrum of compound **10.**

Figure S13. ^{1}H-NMR spectrum of compound **10** (400 MHz, $CDCl_3$).

Figure S14. ^{13}C-NMR spectrum of compound **10** (125 MHz, $CDCl_3$).

Figure S15. DEPT spectrum of compound **10** (135 MHz, $CDCl_3$).

Figure S16. HSQC spectrum of compound **10** (400 MHz, $CDCl_3$).

Figure S17. COSY spectrum of compound **10** (400 MHz, $CDCl_3$).

Figure S18. HMBC spectrum of compound **10** (400 MHz, $CDCl_3$).

Figure S19. TOCSY spectrum of compound **10** (400 MHz, $CDCl_3$).

Figure S20. NOESY spectrum of compound **10** (400 MHz, $CDCl_3$).

Figure S21. HRESI-MS spectrum of compound **11.**

Figure S22. IR spectrum of compound **11.**

Figure S23. ^{1}H-NMR spectrum of compound **11** (400 MHz, $CDCl_3$).

Figure S24. ^{13}C-NMR spectrum of compound **11** (125 MHz, $CDCl_3$).

Figure S25. DEPT spectrum of compound **11** (135 MHz, $CDCl_3$).

Figure S26. HSQC spectrum of compound **11** (400 MHz, $CDCl_3$).

Figure S27. COSY spectrum of compound **11** (400 MHz, $CDCl_3$).

Figure S28. HMBC spectrum of compound **11** (400 MHz, $CDCl_3$).

Figure S29. TOCSY spectrum of compound **11** (400 MHz, $CDCl_3$).

Figure S30. NOESY spectrum of compound **11** (400 MHz, $CDCl_3$).

Figure S31. Experimental circular dicroism espectrum of **9**.

Figure S32. Experimental circular dicroism espectrum of **10**.

Figure S33. Experimental circular dicroism espectrum of **11**.

Figure S34. Phytochemical screening profile of *C. guatemalensis* by HPLC–ESI–Q–TOF–MS/MS in positive and negative modes.

Figure S35. Concentration-response inhibition curves of G6Pase.

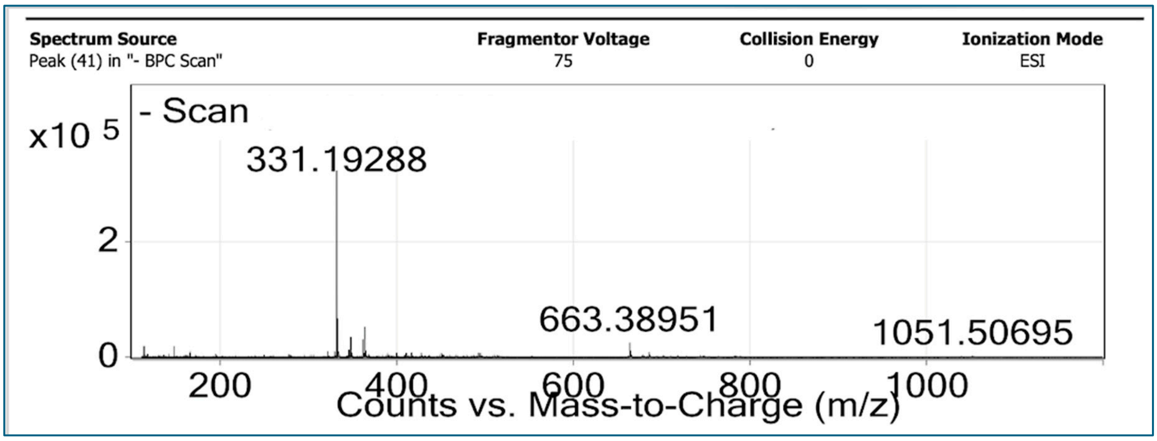

Figure S1. HRESI-MS spectrum of compound **9.**

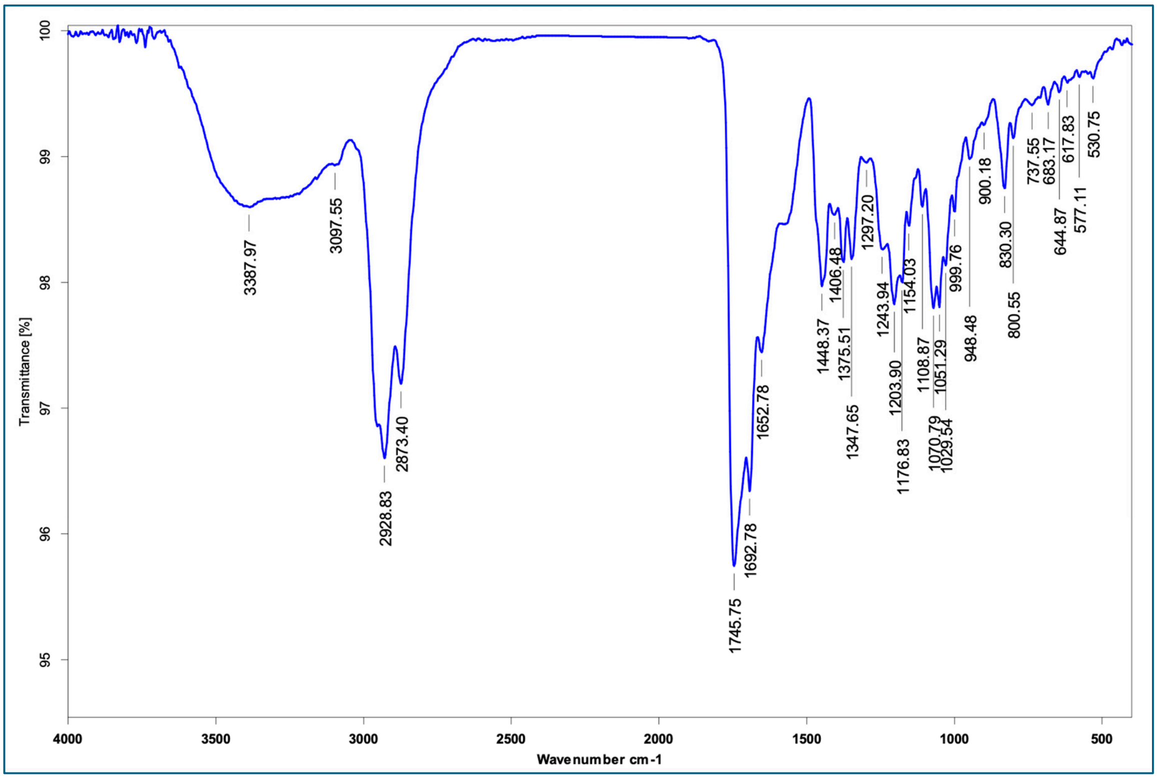

Figure S2. IR spectrum of compound **9.**

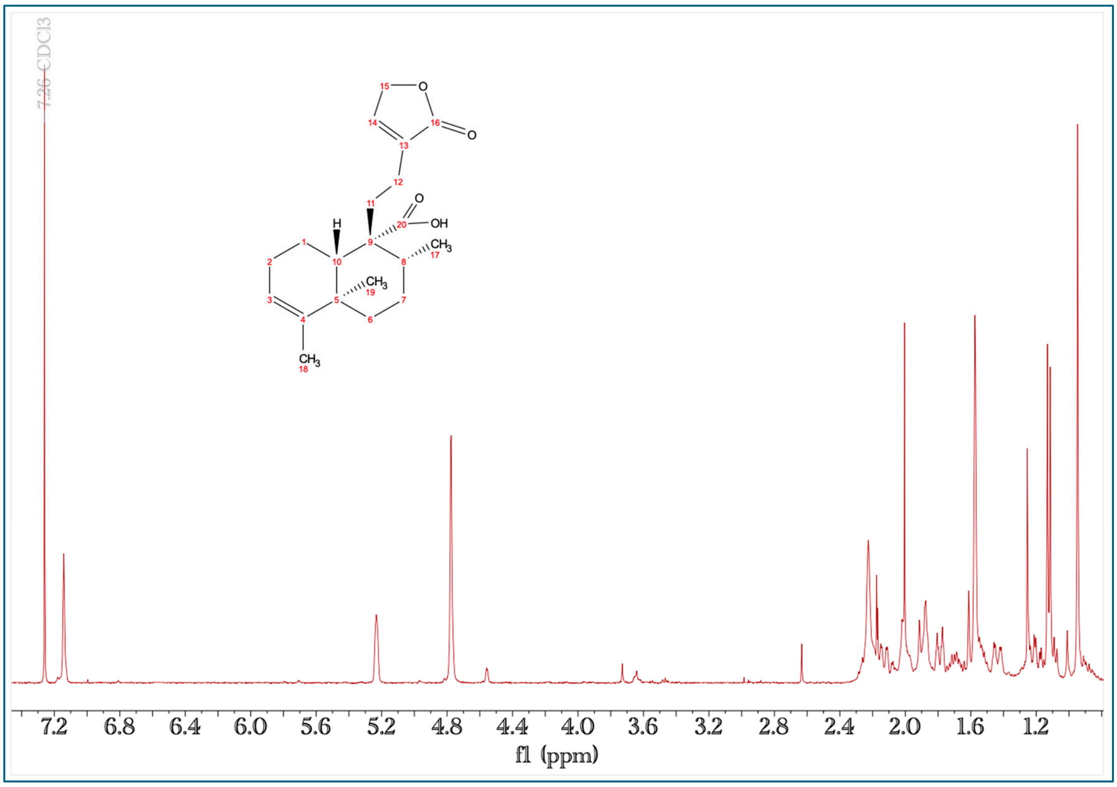

Figure S3. ^{1}H-NMR spectrum of compound **9** (400 MHz, $CDCl_3$).

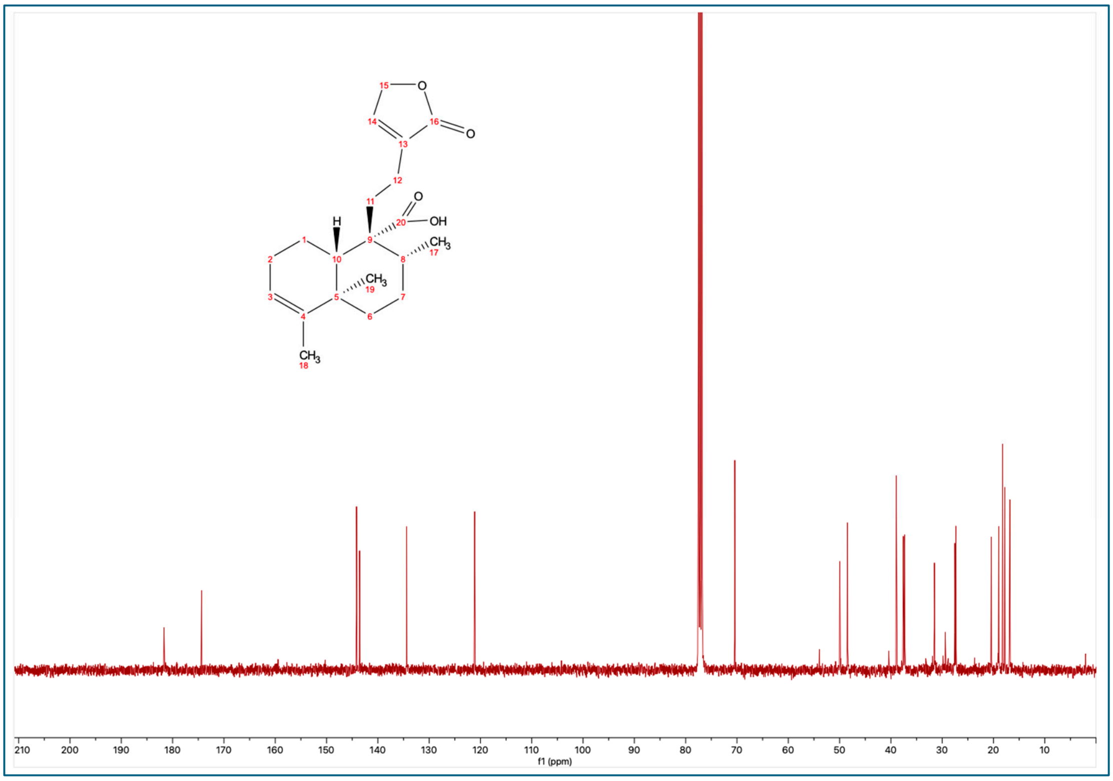

Figure S4. ^{13}C-NMR spectrum of compound **9** (125 MHz, $CDCl_3$).

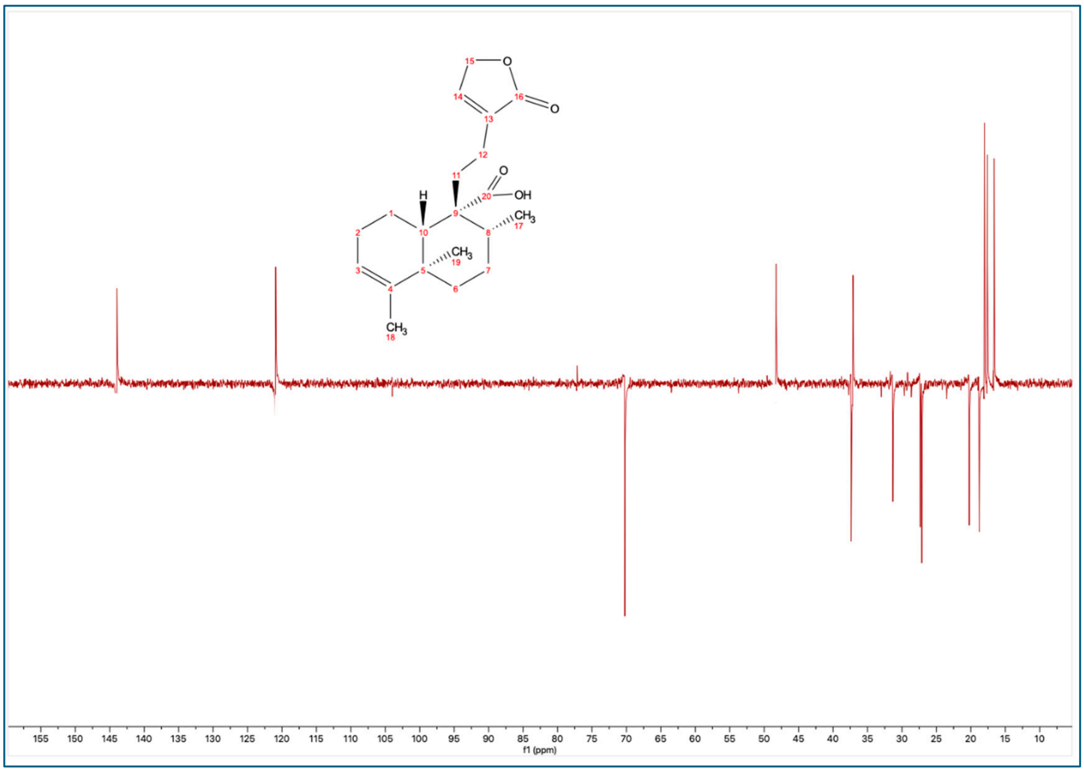

Figure S5. DEPT spectrum of compound **9** (135 MHz, $CDCl_3$).

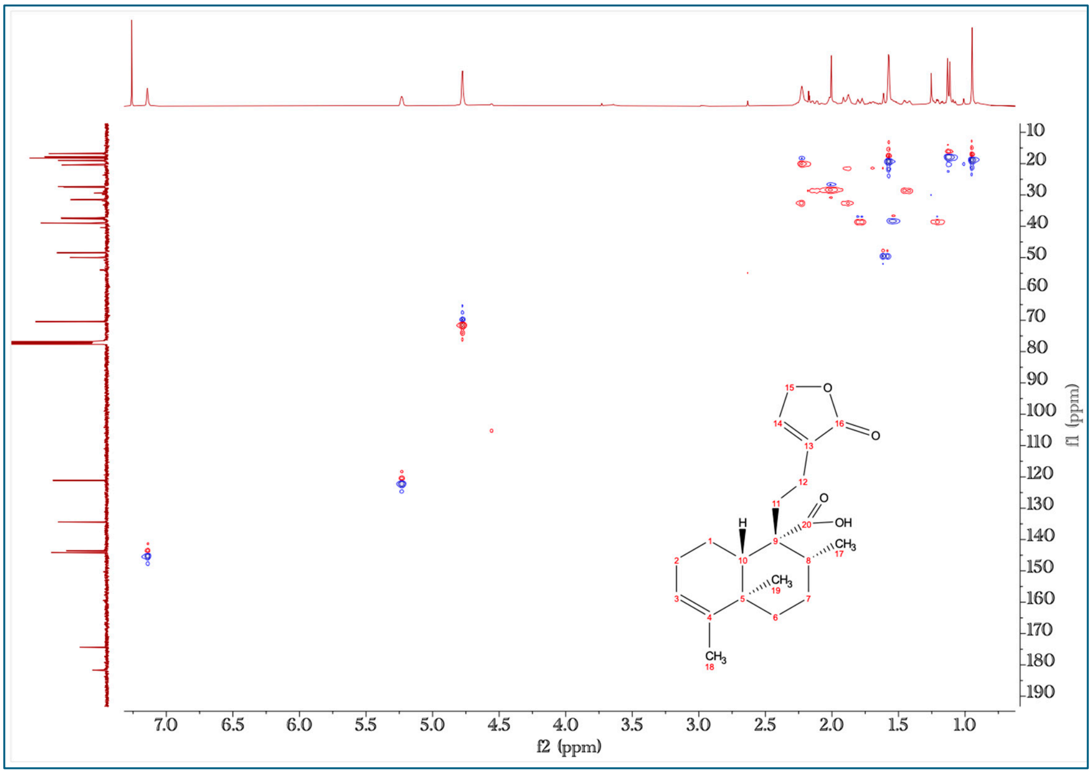

Figure S6. HSQC spectrum of compound **9** (400 MHz, $CDCl_3$).

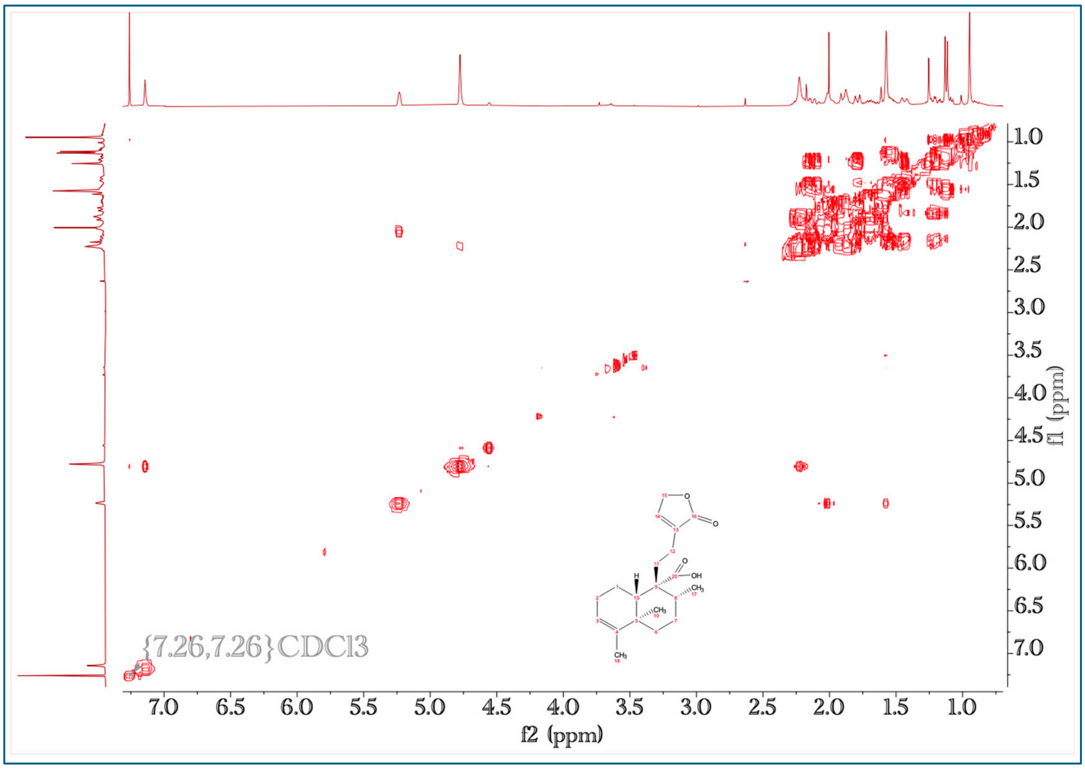

Figure S7. COSY spectrum of compound **9** (400 MHz, $CDCl_3$).

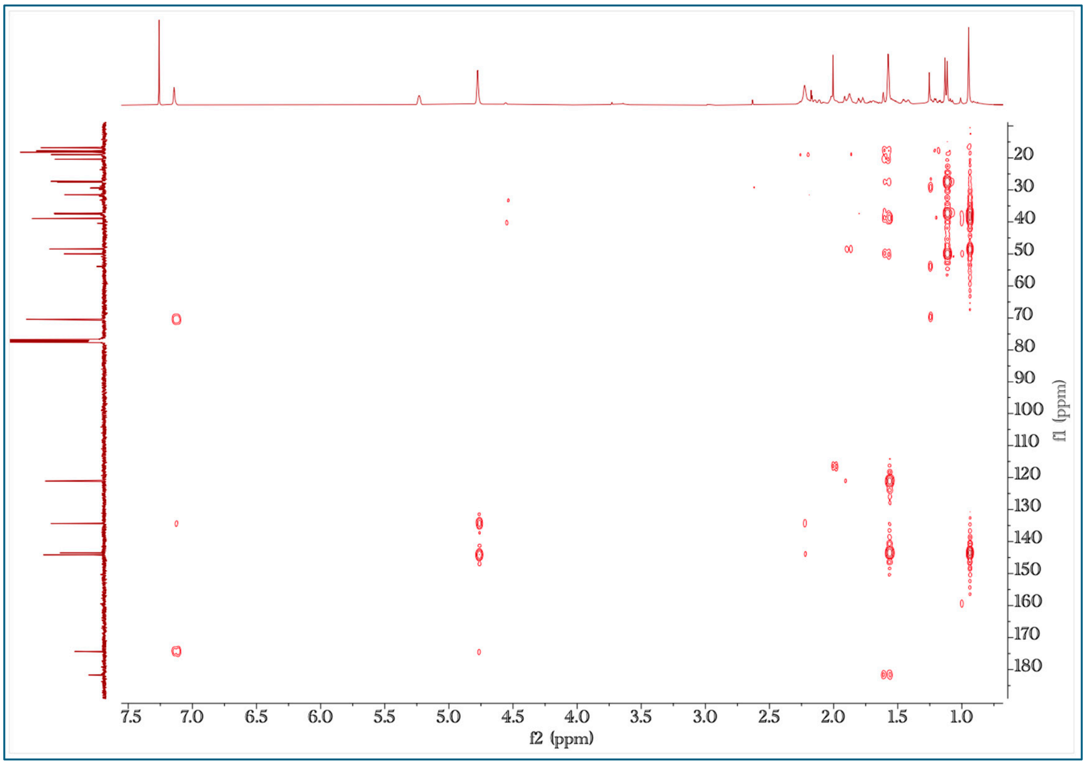

Figure S8. HMBC spectrum of compound **9** (400 MHz, $CDCl_3$).

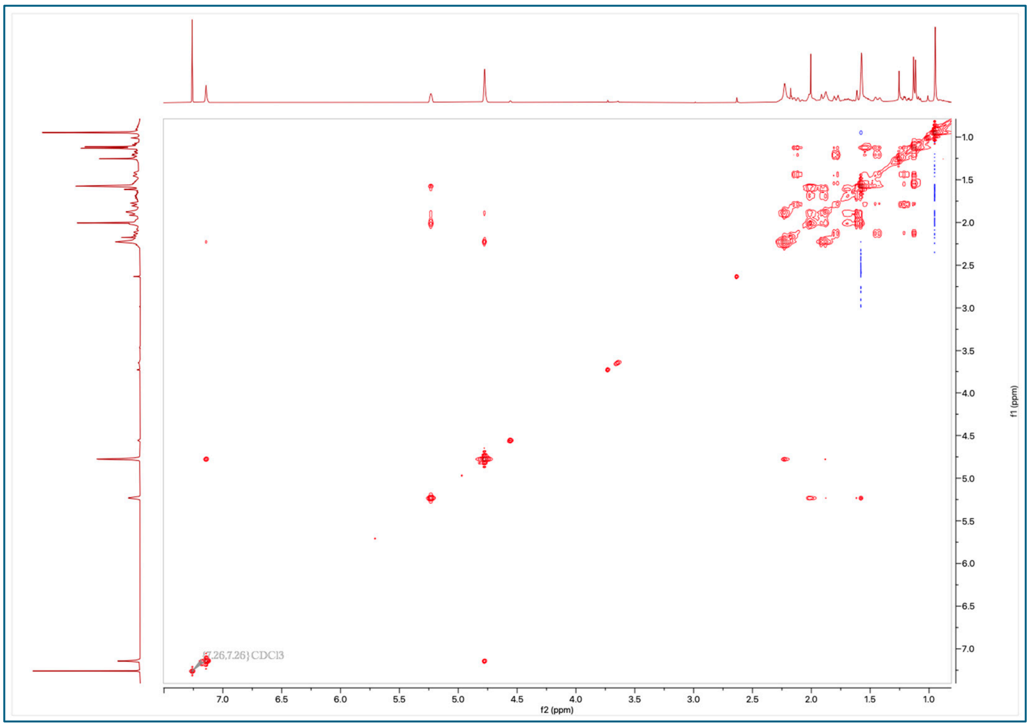

Figure S9. TOCSY spectrum of compound **9** (400 MHz, $CDCl_3$).

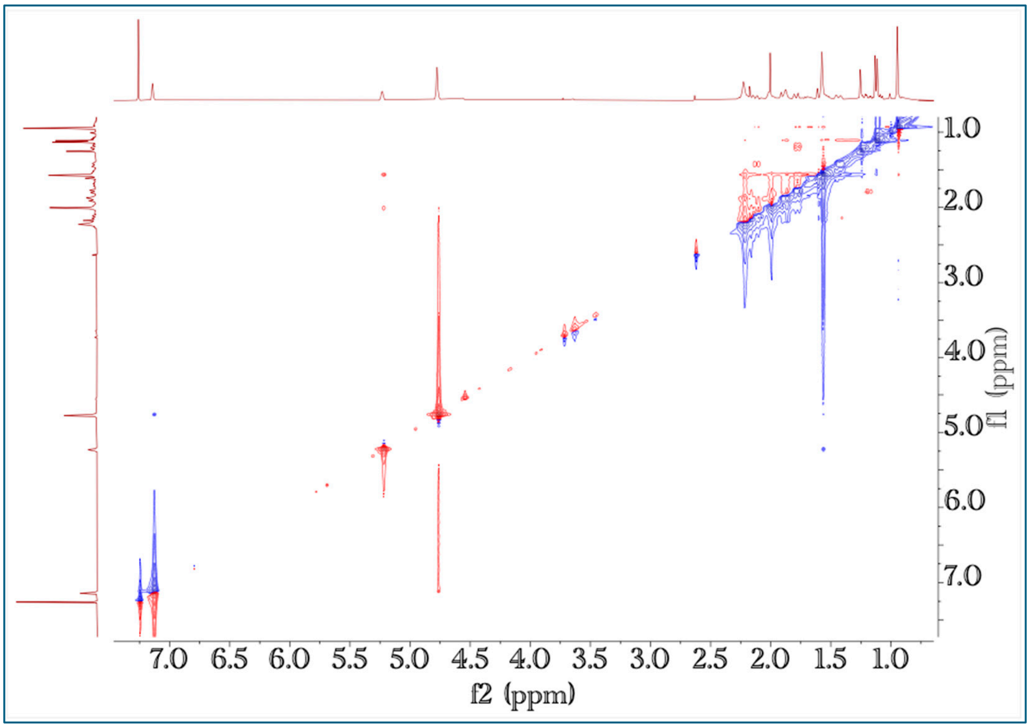

Figure S10. NOESY spectrum of compound **9** (400 MHz, $CDCl_3$).

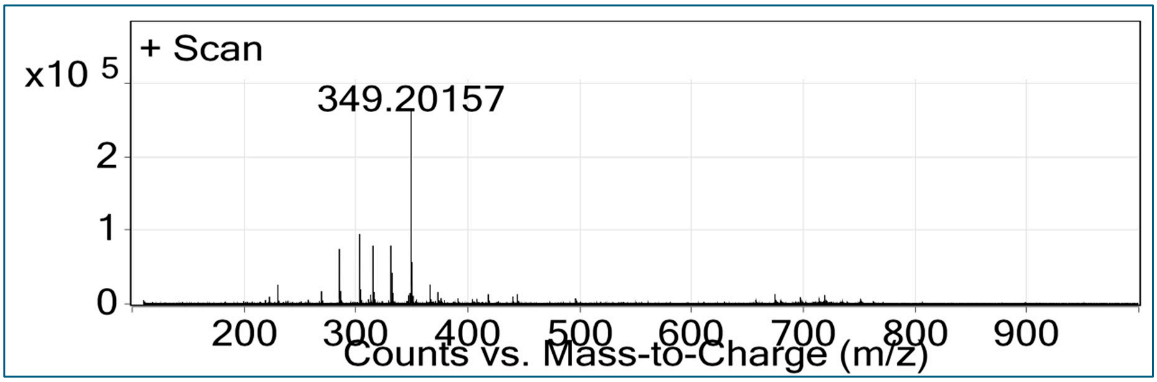

Figure S11. HRESI-MS spectrum of compound **10.**

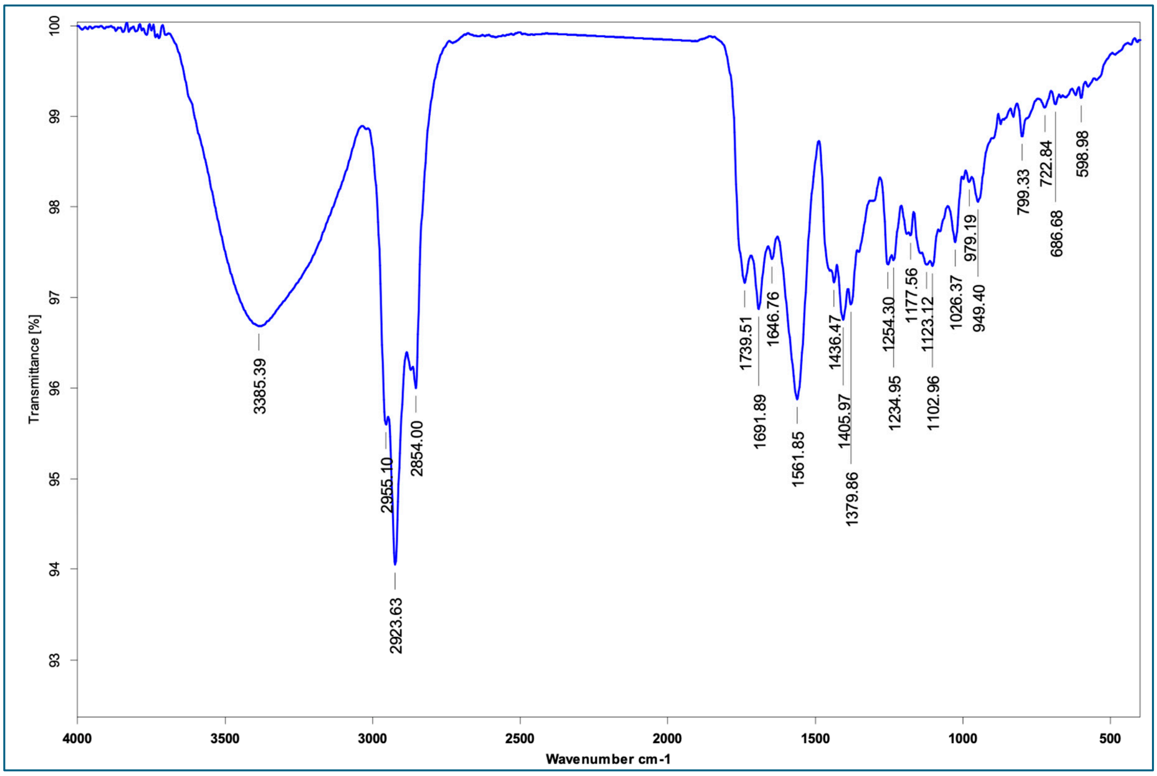

Figure S12. IR spectrum of compound **10.**

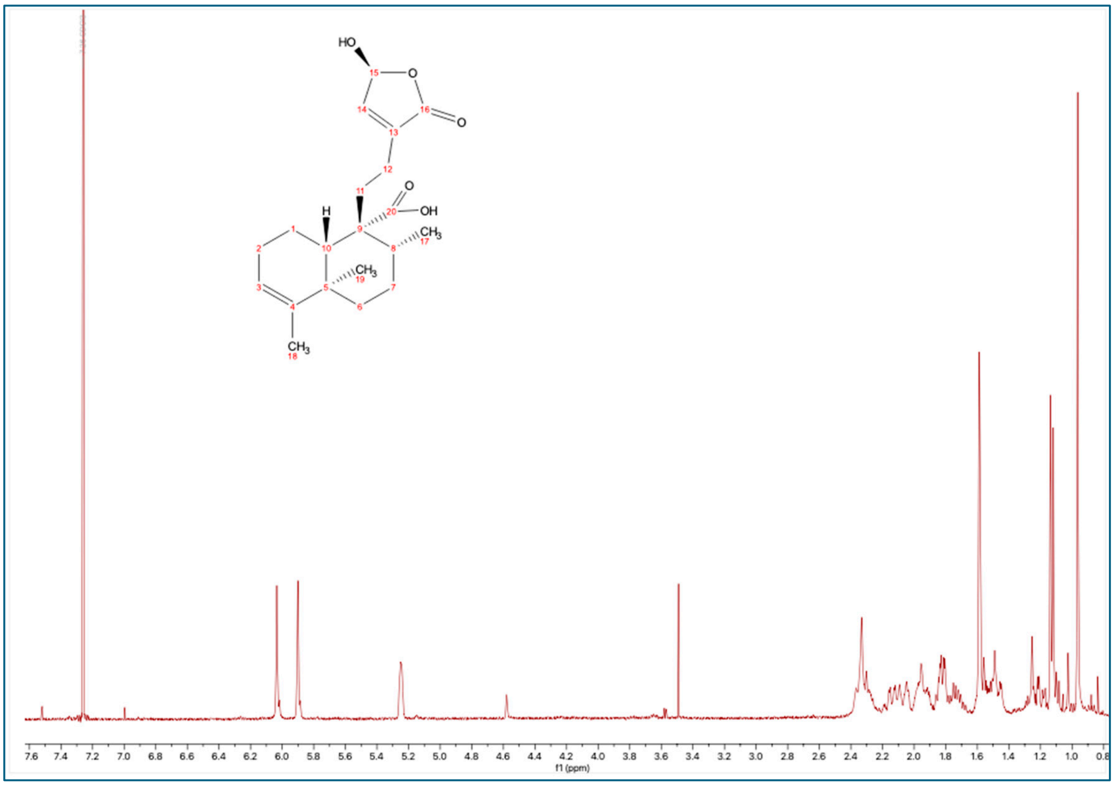

Figure S13. ^{1}H-NMR spectrum of compound **10** (400 MHz, $CDCl_3$).

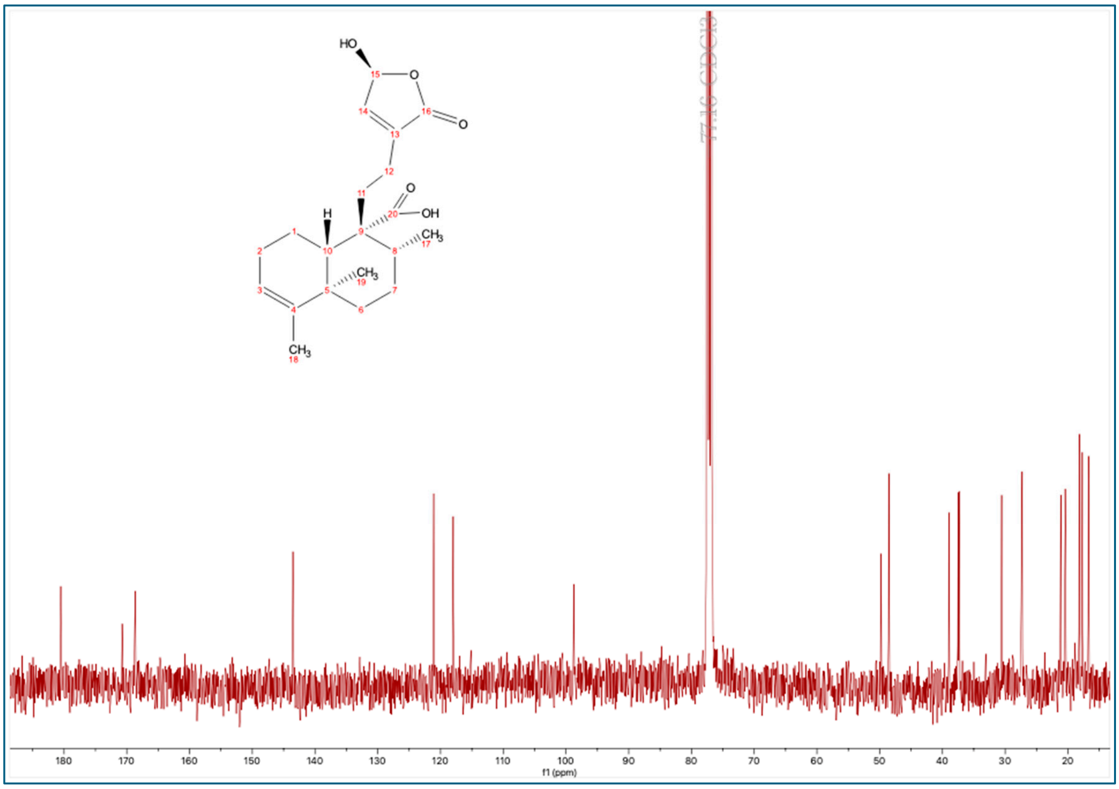

Figure S14. ^{13}C-NMR spectrum of compound **10** (125 MHz, $CDCl_3$).

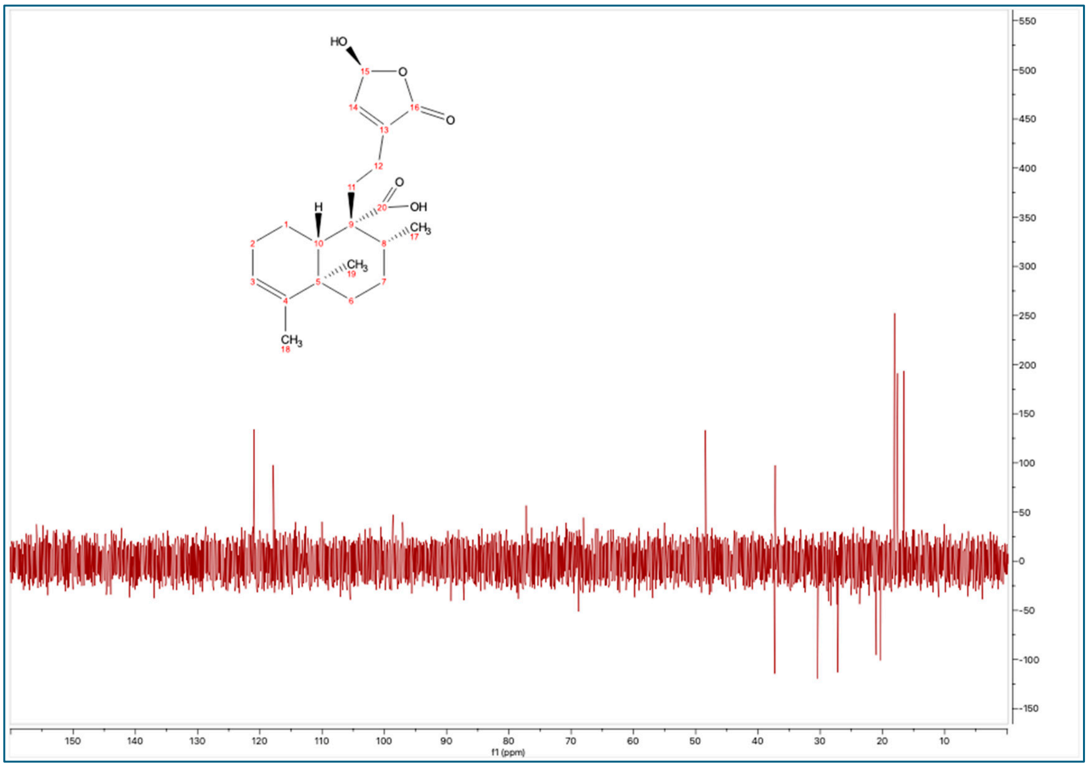

Figure S15. DEPT spectrum of compound **10** (135 MHz, $CDCl_3$).

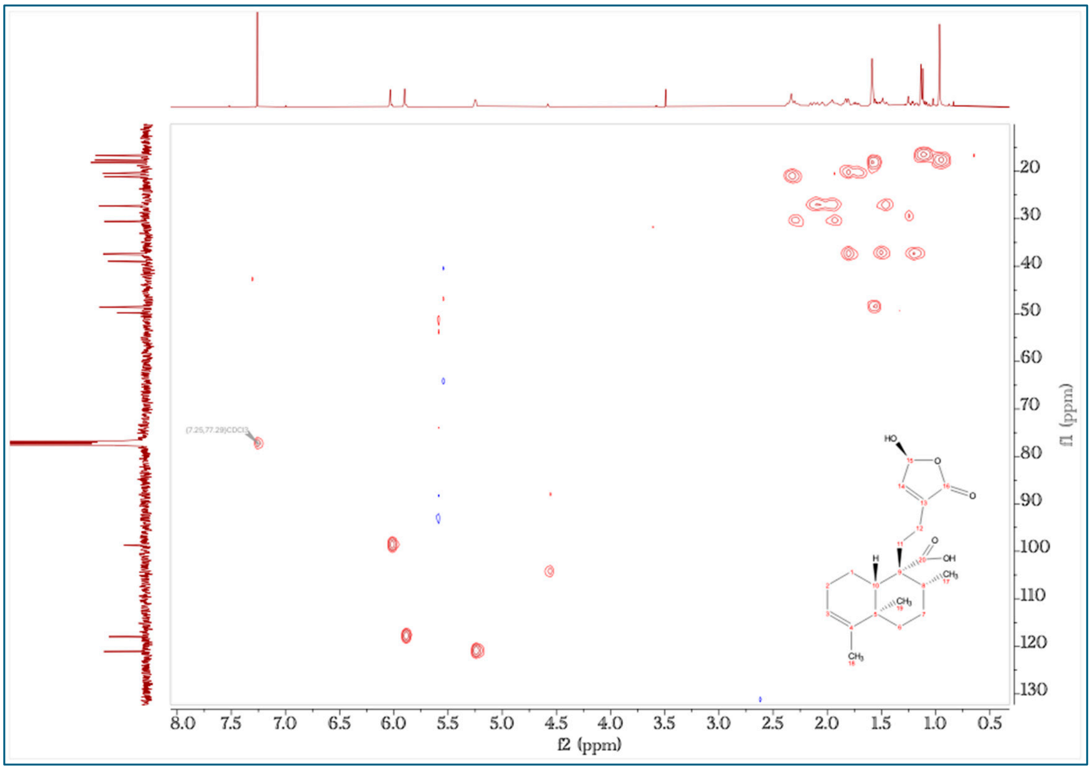

Figure S16. HSQC spectrum of compound **10** (400 MHz, $CDCl_3$).

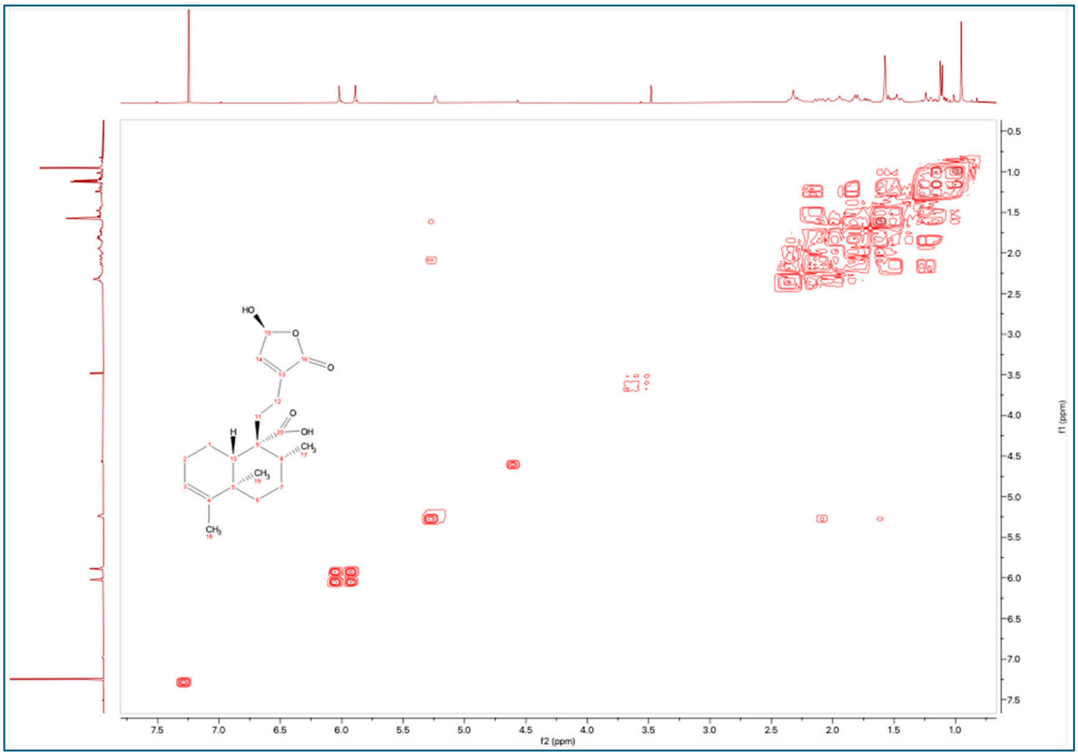

Figure S17. COSY spectrum of compound **10** (400 MHz, $CDCl_3$).

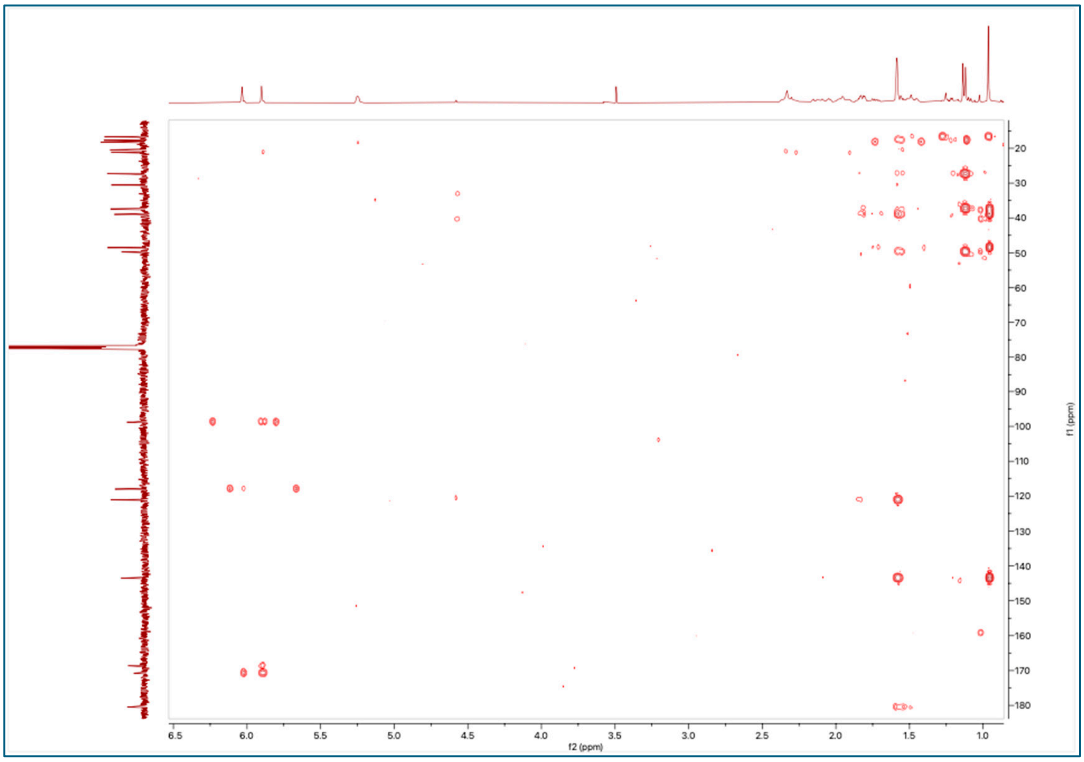

Figure S18. HMBC spectrum of compound **10** (400 MHz, $CDCl_3$).

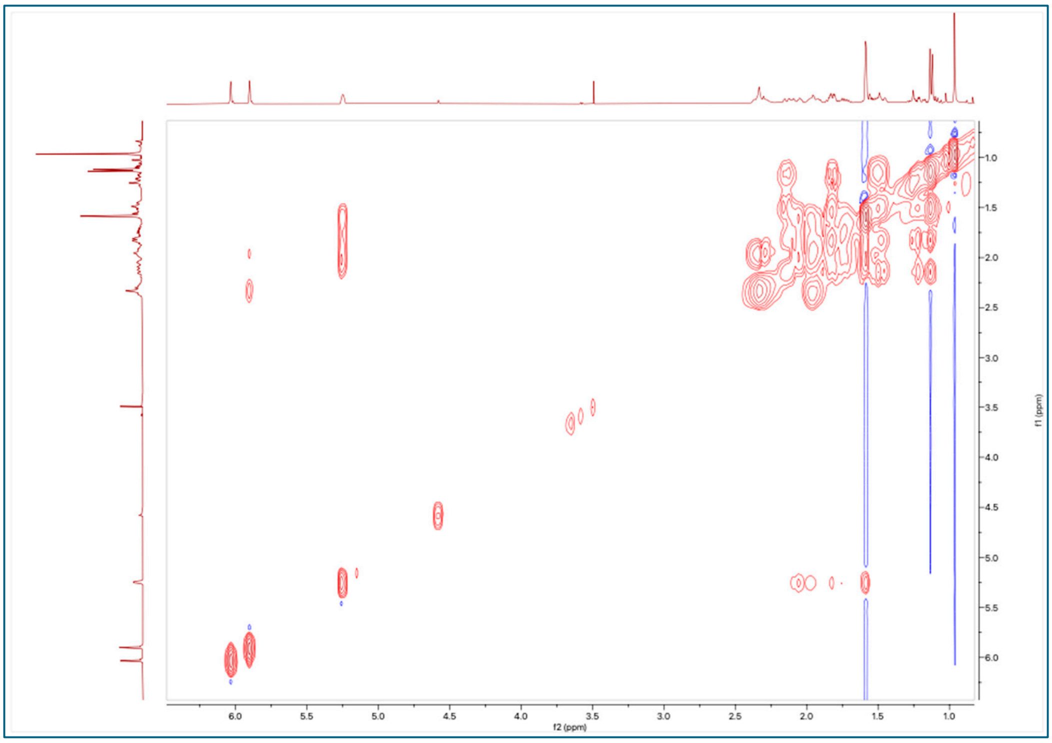

Figure S19. TOCSY spectrum of compound **10** (400 MHz, $CDCl_3$).

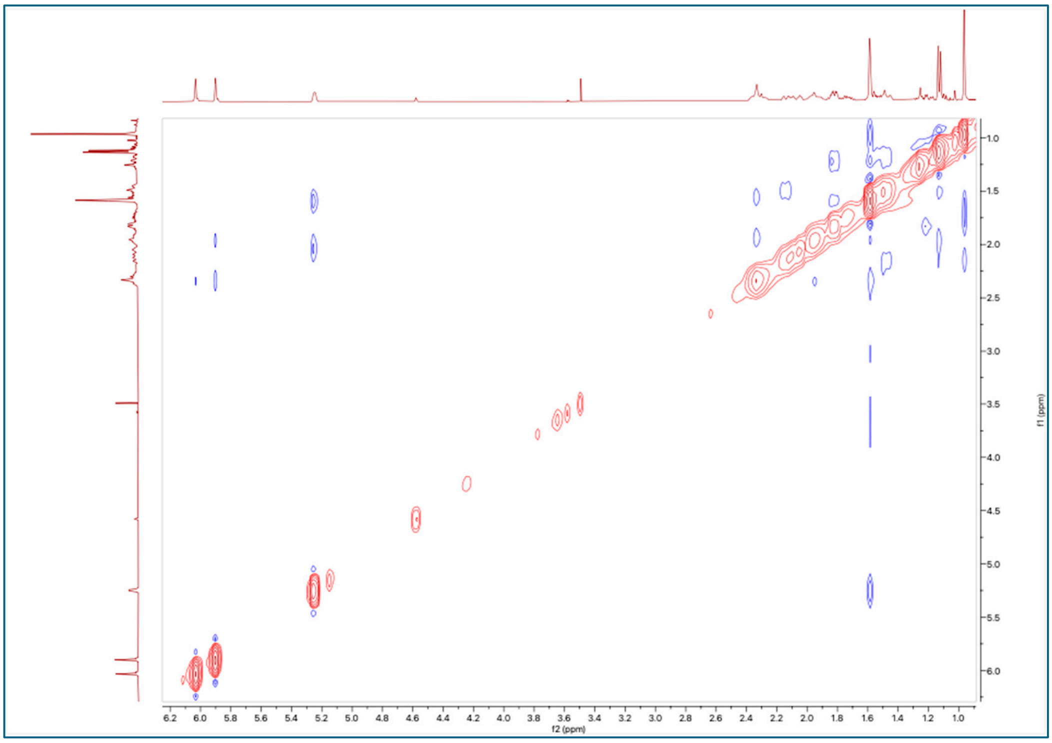

Figure S20. NOESY spectrum of compound **10** (400 MHz, $CDCl_3$).

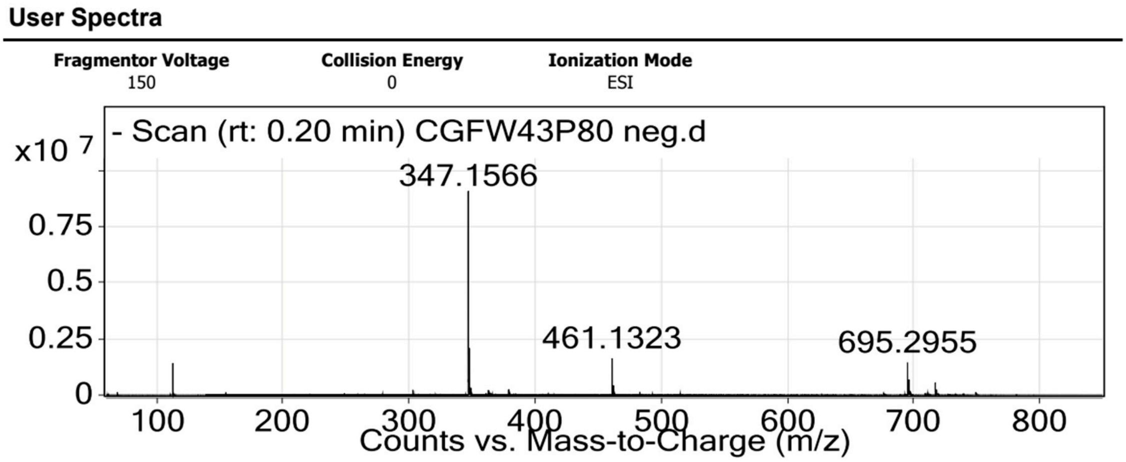

Figure S21. HRESI-MS spectrum of compound **11.**

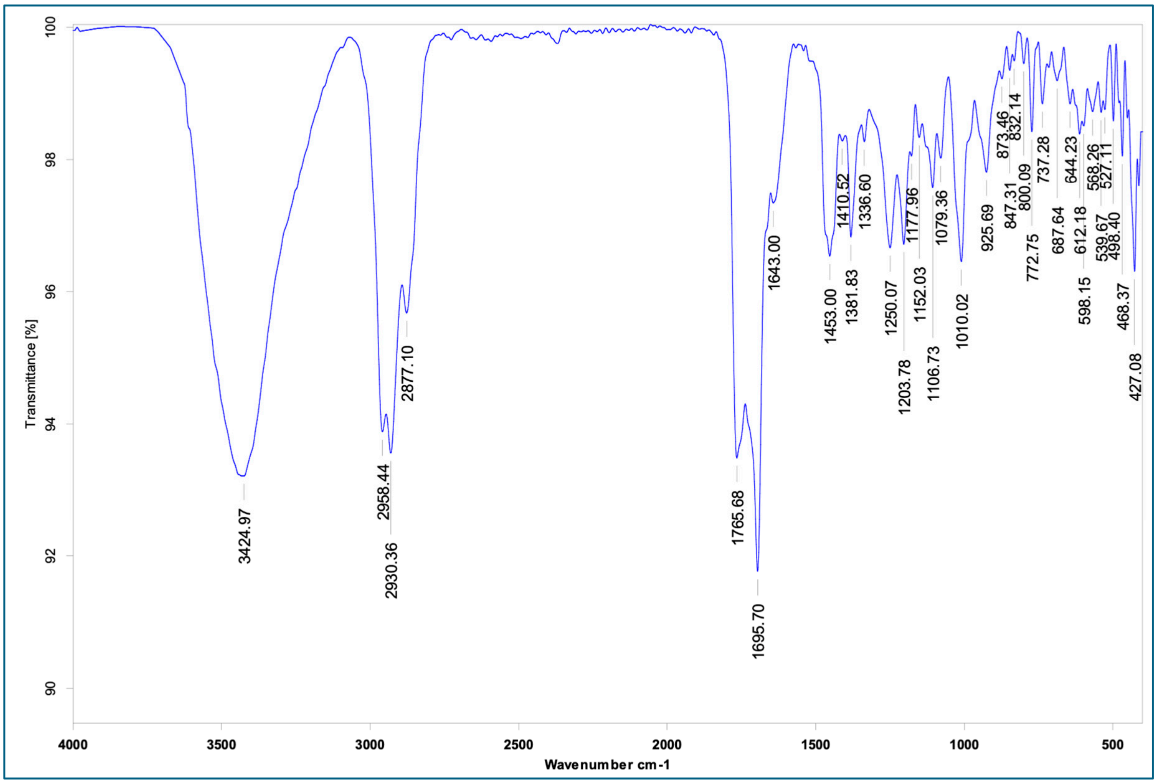

Figure S22. IR spectrum of compound **11.**

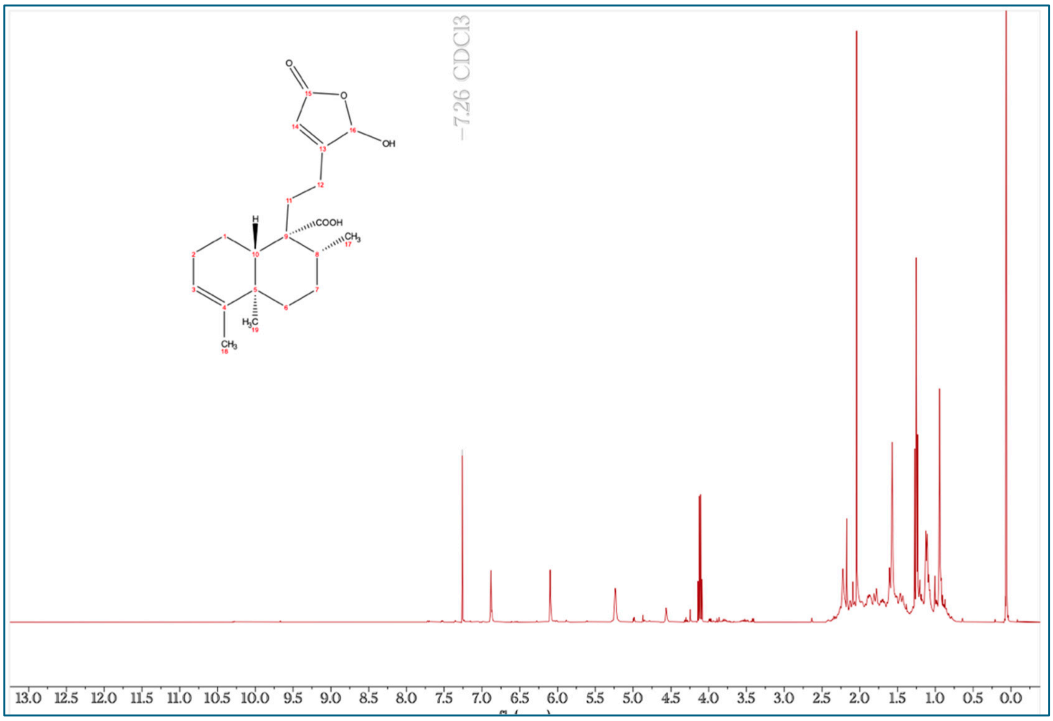

Figure S23. ^{1}H-NMR spectrum of compound **11** (400 MHz, $CDCl_3$).

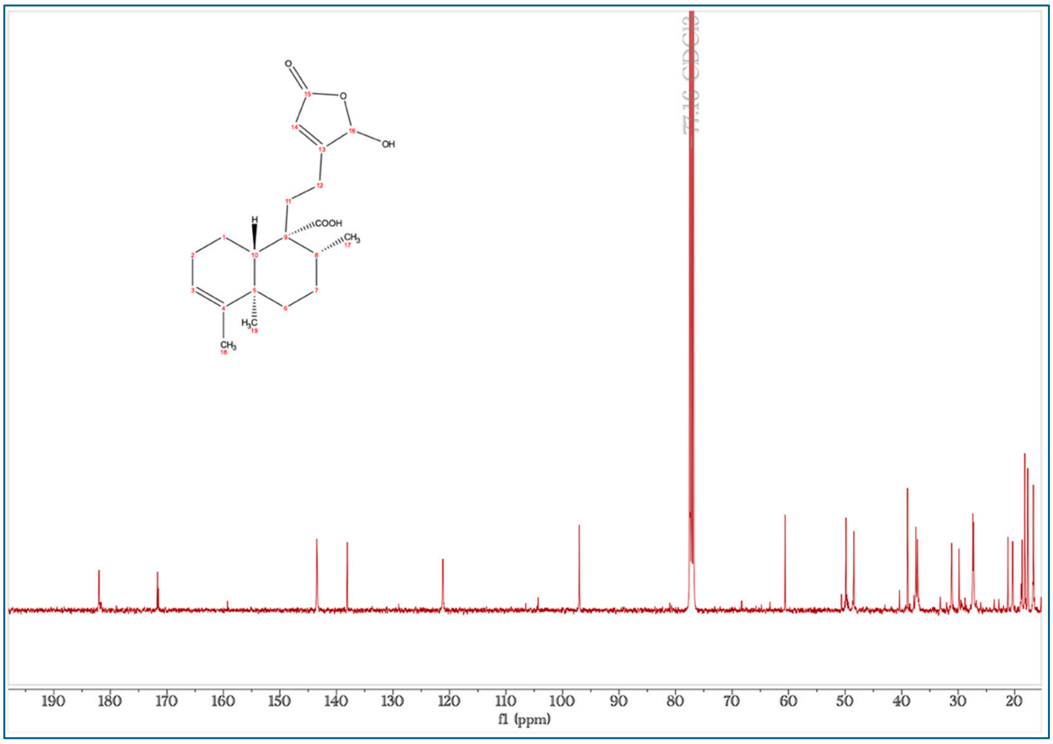

Figure S24. ^{13}C-NMR spectrum of compound **11** (125 MHz, $CDCl_3$).

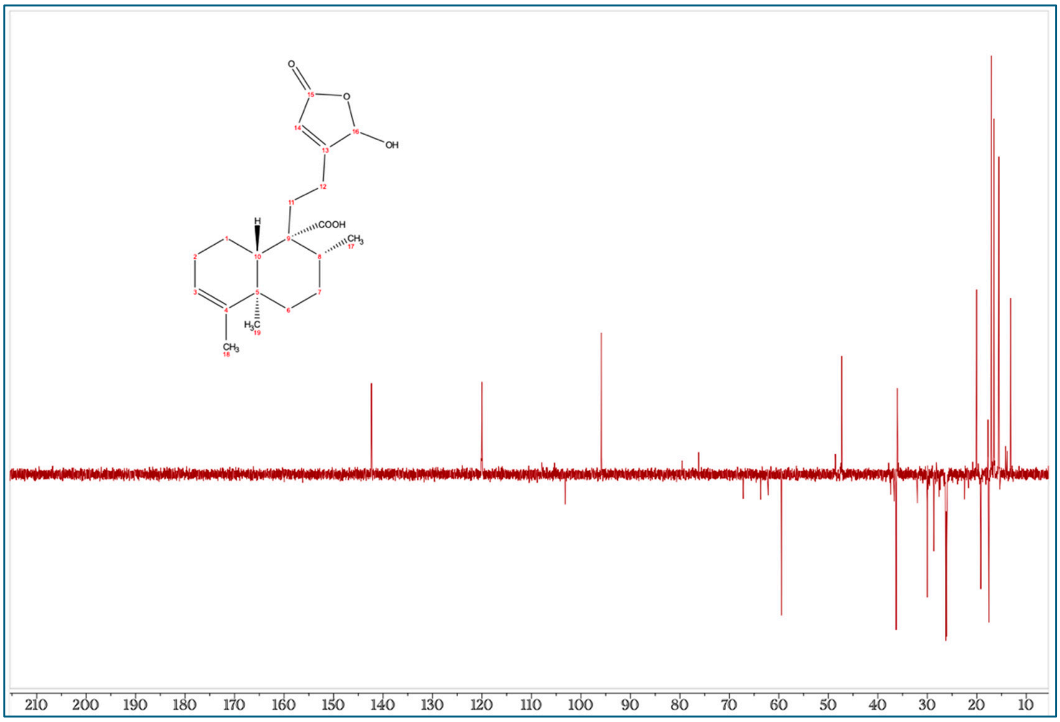

Figure S25. DEPT spectrum of compound **11** (135 MHz, $CDCl_3$).

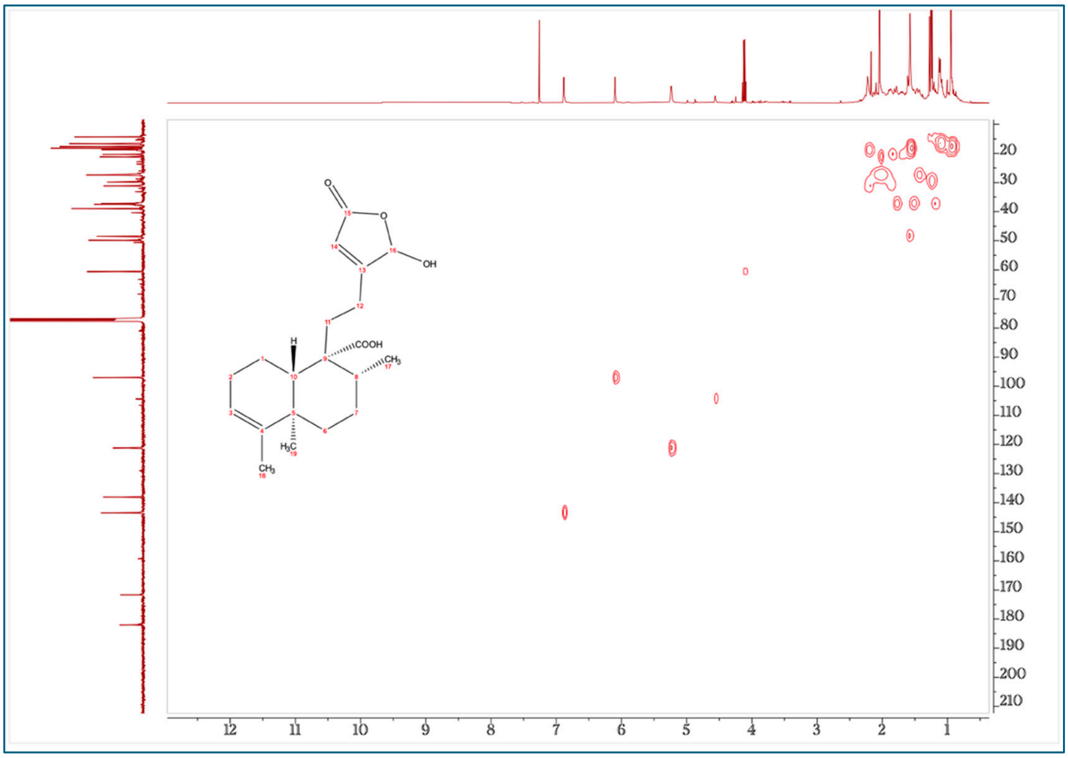

Figure S26. HSQC spectrum of compound **11** (400 MHz, $CDCl_3$).

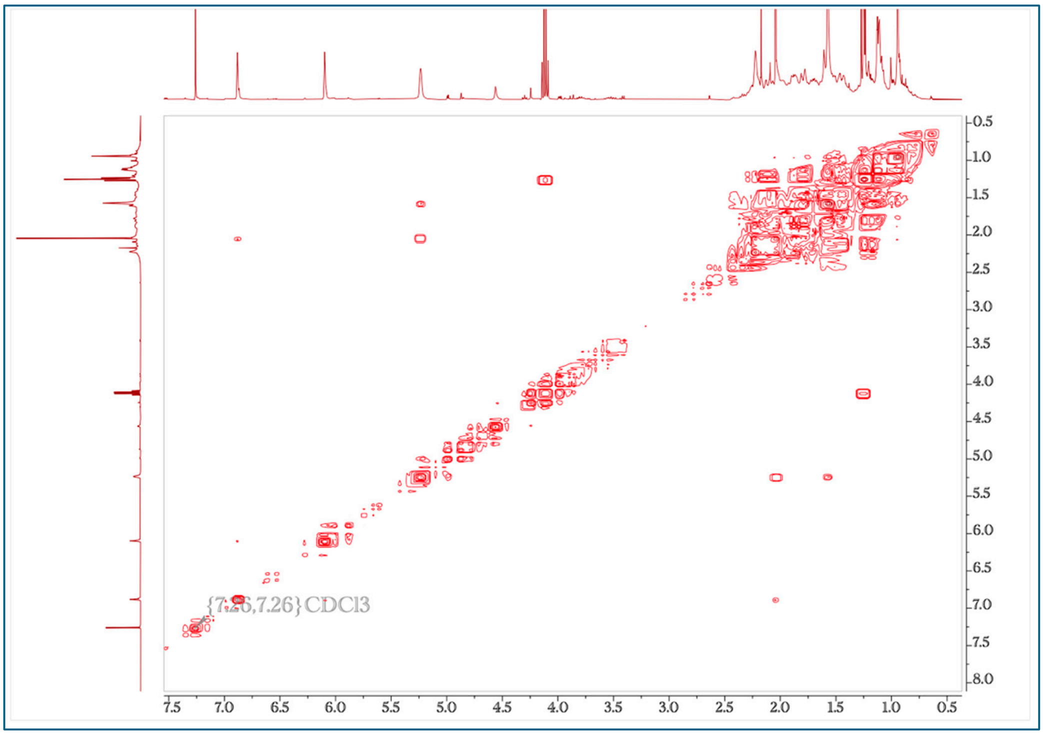

Figure S27. COSY spectrum of compound **11** (400 MHz, $CDCl_3$).

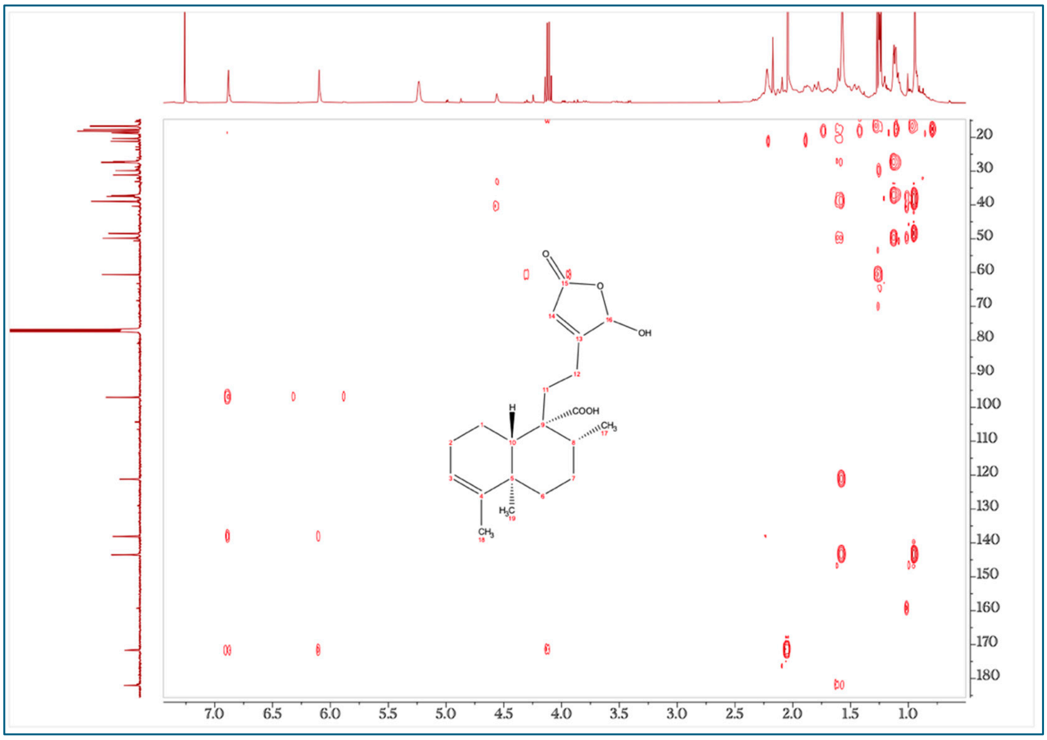

Figure S28. HMBC spectrum of compound **11** (400 MHz, $CDCl_3$).

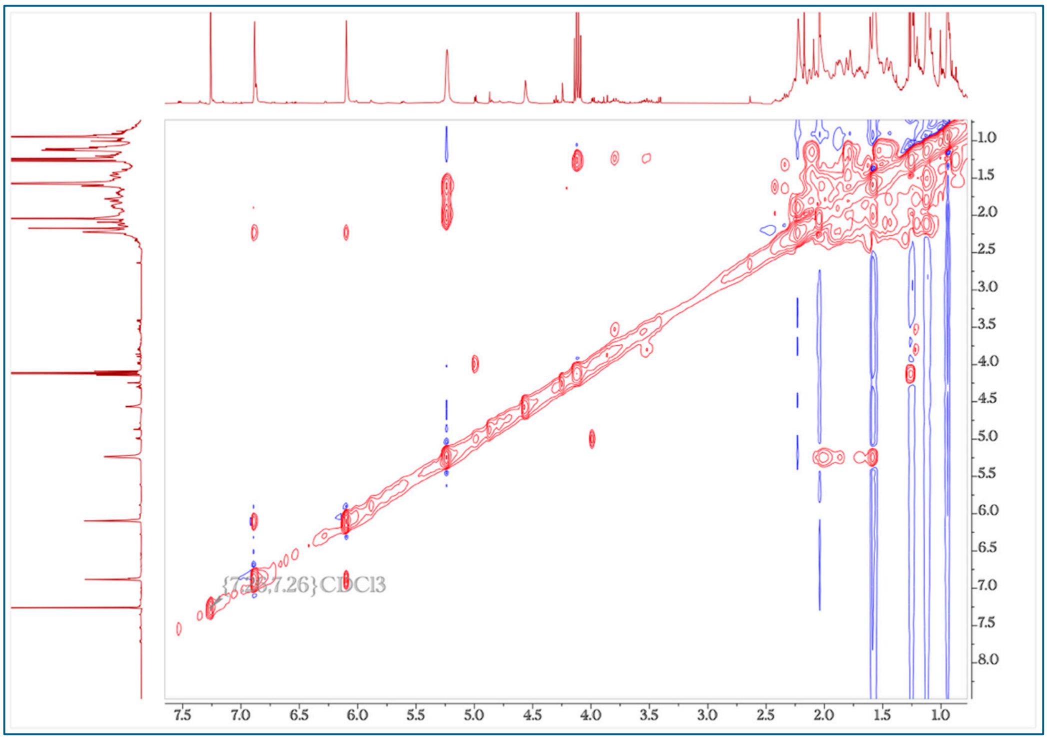

Figure S29. TOCSY spectrum of compound **11** (400 MHz, $CDCl_3$).

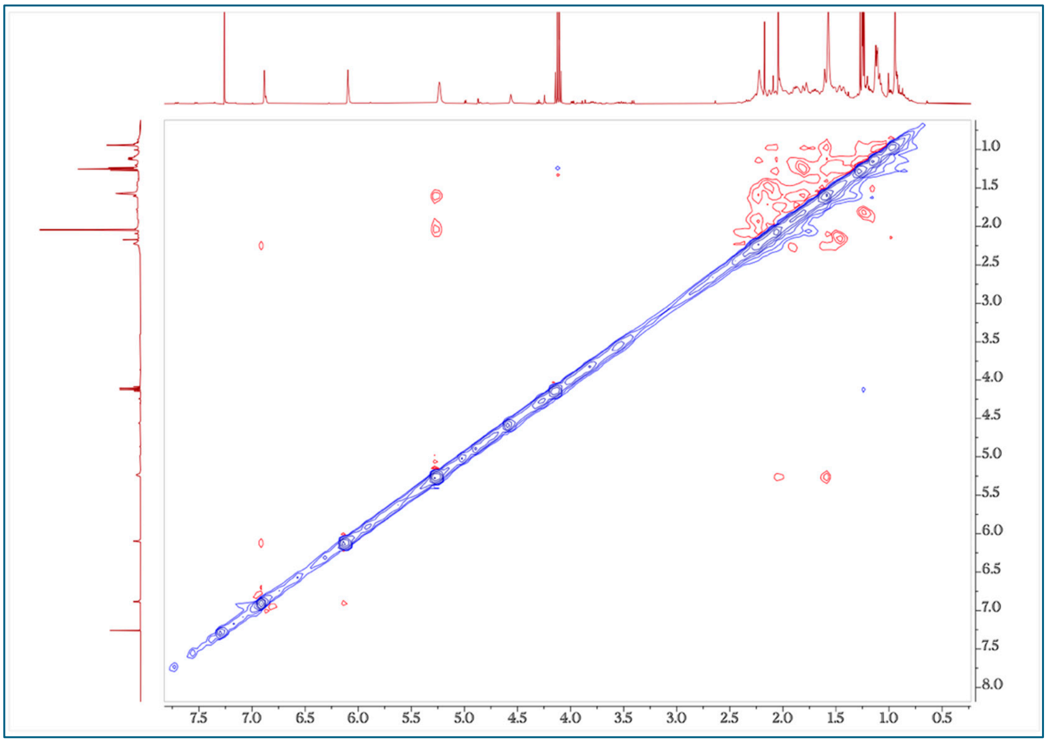

Figure S30. ROESY spectrum of compound **11** (400 MHz, $CDCl_3$).

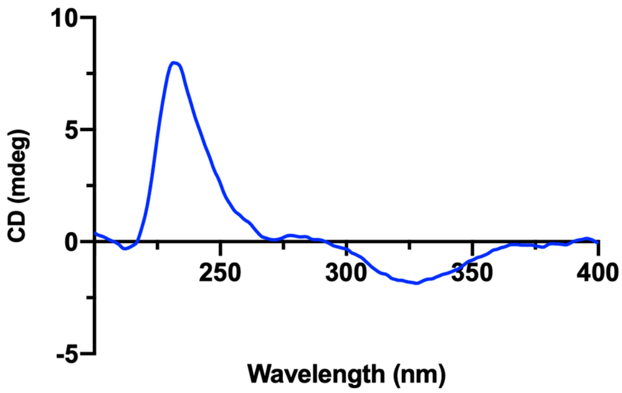

Figure 31. Experimental circular dicroism espectrum of **9**.

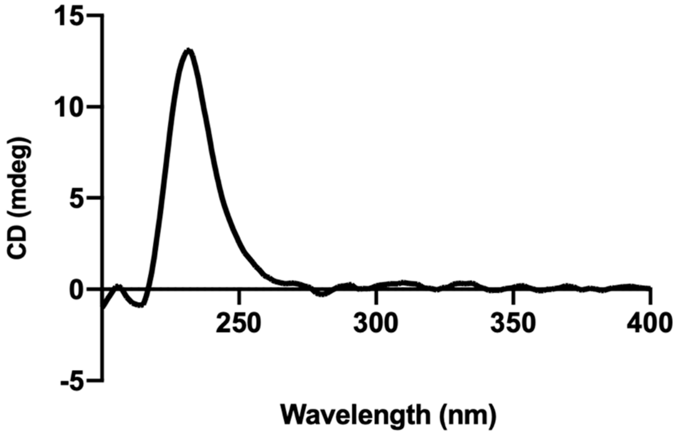

Figure 32. Experimental circular dicroism espectrum of **10**.

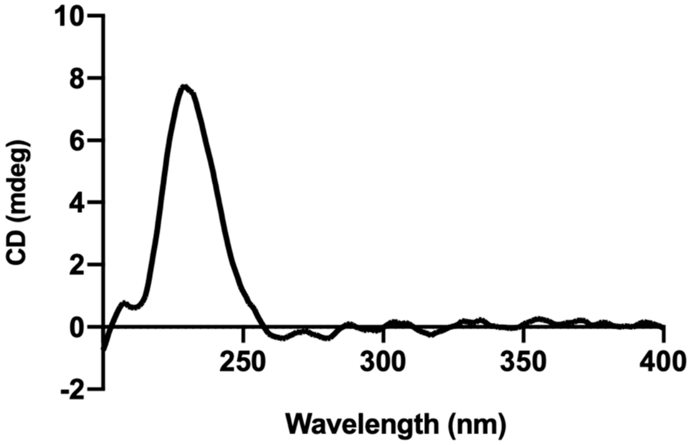

Figure 33. Experimental circular dicroism espectrum of **11**.

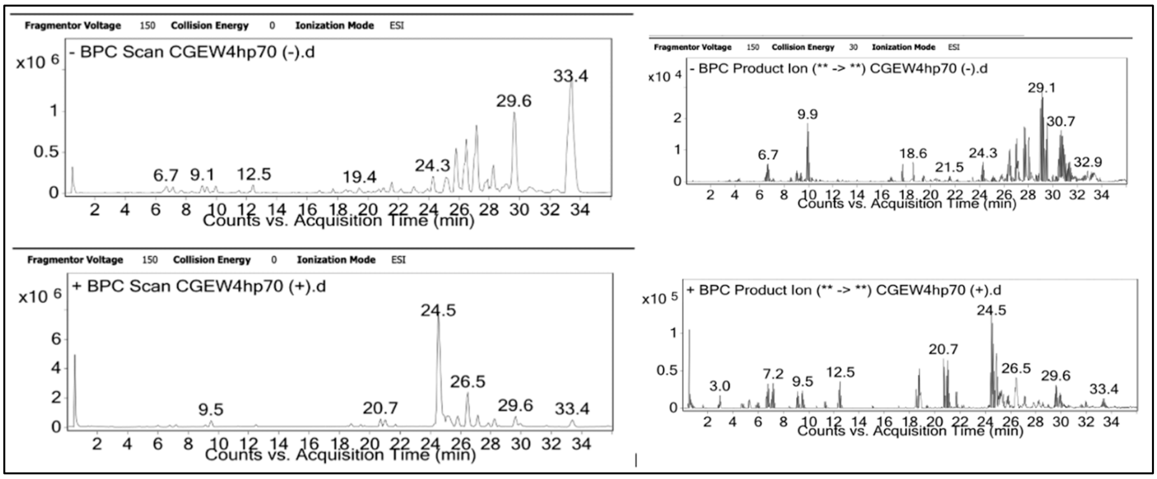

Figure S34. Phytochemical screening profile of *C. guatemalensis* by HPLC–ESI–QTOF–MS/MS in positive and negative modes.

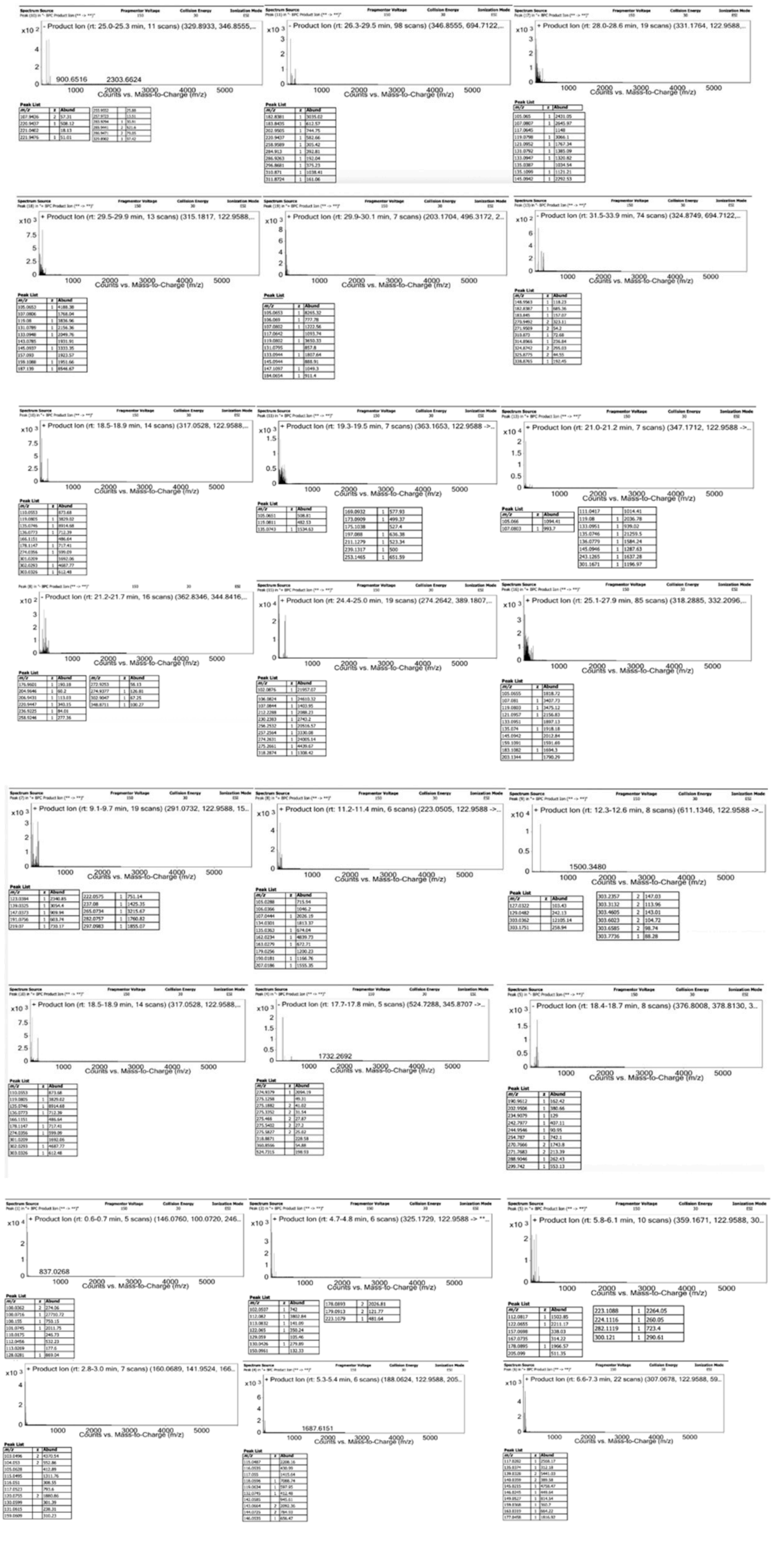

Figure S35. MS/MS spectra of the HPLC-MS profile of the ethanol:water extract of C. guatemalensis

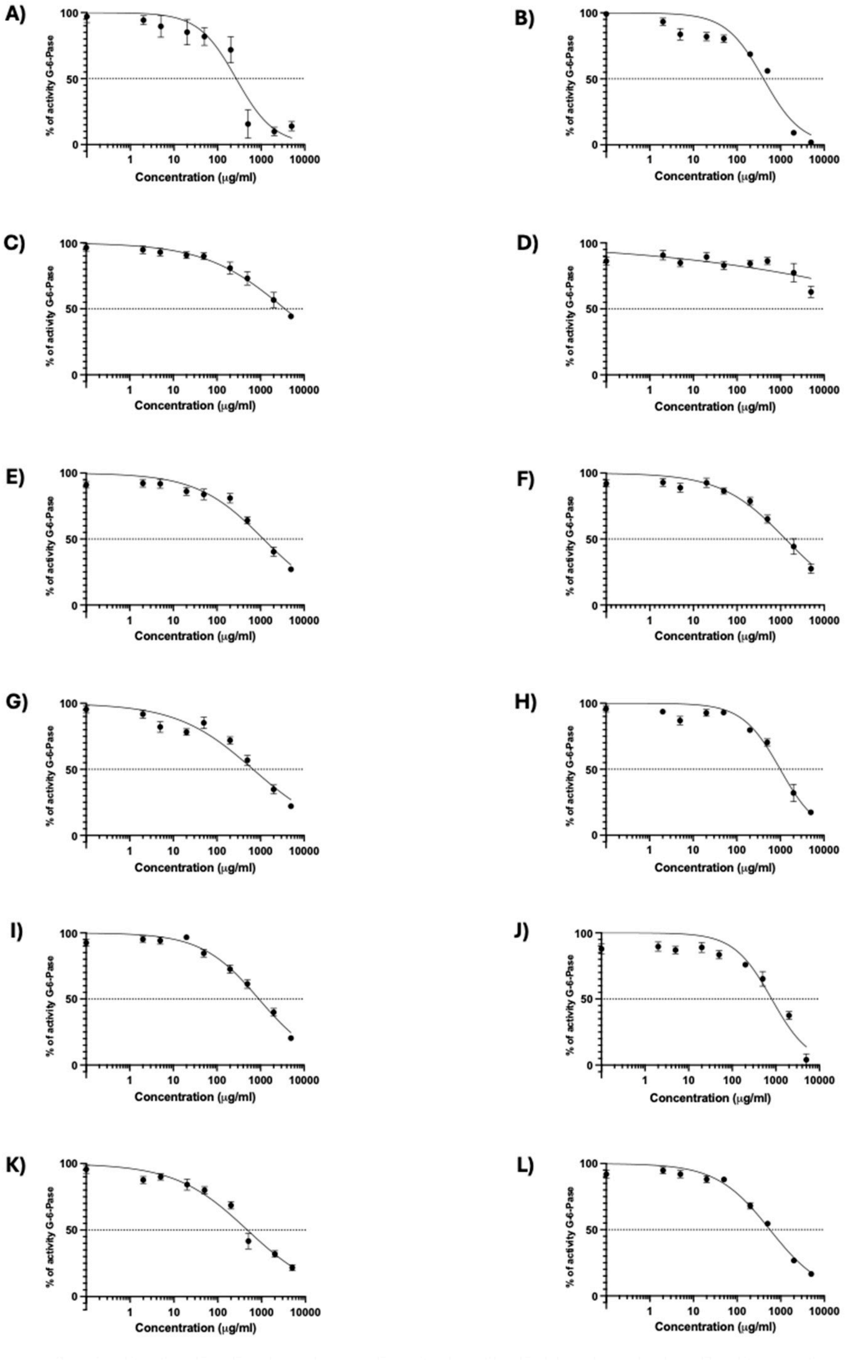

Figure S36. Concentration-response inhibition curves of G6Pase. **A)** Extract *C. guatemalensis* **B)** Chlorogenic acid, **C)** epicatechin, **D)** Rutin, **E)** Bartsiifolic acid, **F)** Crotoguatenoic acid A, **G)** Crotoguatenoic acid B, **H)** Crotoguatenoic acid C, **I)** Crotoguatenoic acid D, **J)** Crotoguatenoic acid E, **K)** Formosin, **L)** Junceic acid. Each point represents the mean ± SEM.
